# Influence of tillage and residue management practices on productivity, sustainability, and soil biological properties of rice-barley cropping systems in indo-gangetic plain of India

**DOI:** 10.3389/fmicb.2023.1130397

**Published:** 2023-03-16

**Authors:** Priyanka Chandra, Anil Kumar Khippal, Kailash Prajapat, Arijit Barman, Geeta Singh, Arvind Kumar Rai, Om Parkash Ahlawat, R. P. S. Verma, Kamini Kumari, Gyanendra Singh

**Affiliations:** ^1^Department of Soil and Crop Management, ICAR-Central Soil Salinity Research Institute, Karnal, India; ^2^Barley Network, ICAR-Indian Institute of Wheat and Barley Research, Karnal, India; ^3^Department of Social Science Research, ICAR-Central Soil Salinity Research Institute, Karnal, India; ^4^Department of Agricultural Microbiology, ICAR-Indian Agricultural Research Institute, New Delhi, India; ^5^Department of Crop Improvement, ICAR-Indian Institute of Wheat and Barley Research, Karnal, India; ^6^Department of Soil Science and Agricultural Chemistry, Lovely Professional University, Phagwara, India; ^7^ICAR-Indian Institute of Wheat and Barley Research, Karnal, India

**Keywords:** conservation agriculture, principal component analysis, soil biological properties, soil biological index, system productivity

## Abstract

**Introduction:**

Conservation agriculture is a sustainable system of farming that safeguard and conserves natural resources besides enhancing crop production. The biological properties of soil are the most sensitive indicator to assess the short term impact of management practices such as tillage and residue incorporation.

**Methods:**

Nine treatments of tillage and residue management practices [Reduced till direct seeded rice-zero till barley (RTDSR–ZTB); RTDSR–ZTB–green gram residue (Gg); Zero till direct seeded rice–zero till barley–zero till green gram (ZTDSR–ZTB–ZTGg); RTDSR–ZTB + rice residue at 4 t ha 1 (RTDSR–ZTB_RR4_); RTDSR–ZTB_RR6_; un-puddled transplanted rice (UPTR)–ZTB–Gg; UPTR–ZTB_RR4_; UPTR–ZTB_RR6_, and puddled transplanted rice (PTR)–RTB] executed under fixed plot for five years on crop productivity and soil biological properties under rice-barley production system.

**Results:**

The shifting in either RTDSR or ZTDSR resulted in yield penalty in rice compared to PTR. The PTR recorded highest pooled grain yield of 3.61 ha^−1^. The rice grain yield reduced about 10.6% under DSR as compared to PTR. The ZTB along with residue treatments exhibited significantly higher grain yield over ZTB, and the RTDSR-ZTBRR6 registered highest pooled grain yield of barley. The system productivity (12.45 t ha^−1^) and sustainable yield index (0.87) were highest under UPTR-ZTBRR6. Biological parameters including microbial biomass carbon, soil respiration, microbial enzymes (Alkaline phosphatase, nitrate reductase and peroxidase), fluorescein diacetate hydrolysis, ergosterol, glomalin related soil proteins, microbial population (bacteria, fungi and actinobacteria) were found to be significantly (*p* < 0.05) effected by different nutrient management practices. Based on the PCA analysis, Fluorescein diacetate hydrolysis, microbial biomass carbon, soil respiration, nitrate reductase and fungi population were the important soil biological parameters indicating soil quality and productivity in present experiment. The results concluded that UPTR-ZTBRR6 was a more suitable practice for maintaining system productivity and soil biological health.

**Discussion:**

The understanding of the impact of different tillage and residue management practices on productivity, soil biological properties and soil quality index under rice-barley cropping system will help in determining the combination of best conservation agriculture practices for improved soil quality and sustainable production.

## Introduction

The Indo-Gangetic Plain (IGP) of India, spread over 44 million ha, is the most important food-producing region of South Asia. Approximately 76% of its area falls in India, dominated by the states of Punjab, Haryana, Uttar Pradesh, Bihar, and West Bengal (Kumar et al., 2021). The IGP has a wide range of physiographic, climatic, edaphic, and socio-economic production features. The Indian IGP is predominated by a cereal-based production system and contributes to about 40% of the total cereals production of the country.

Rice (*Oryza sativa*)–wheat (*Triticum aestivum* L.) is the main cropping system followed in the IGP covering 13.5 million ha area in Bangladesh, India, Nepal, and Pakistan (Pandey and Kandel, 2020). Despite the fact that the rice–wheat cropping system is the mainstay of the country's food security, its sustainability is at a crossroad due to the development of second-generation problems. It is a high water and nutrient demanding (Ambast et al., 2006; Shahane et al., 2020) crop sequence. The practice of intensive tillage and puddling in standing water for puddled transplanted rice (PTR) results in the breakdown of soil structure, leading to surface and sub-surface soil compaction, poor infiltration and hydraulic conductivity, as well as poor root growth of succeeding crops. Besides, this cropping system leads to a reduction in soil organic matter content, increased greenhouse gas (GHG) emissions (Kakraliya et al., 2021), and a decline in total factor productivity (Kumar et al., 2004).

Other important concerns of this system are the burning of rice residues and the degradation of soil health. Approximately 23 million tons of rice residues are burnt in about 2.5 million farms in the north-western part of India (Meena R.P et al., 2020), which has harmful impacts on the soil and air quality. Large quantities of particulate matter and harmful gases (nitrous oxide, carbon mono oxide, carbon dioxide, methane, etc.) are released into the atmosphere resulting in loss of valuable soil nutrients and deterioration in the air quality leading to ill effects on human health (Jain et al., 2014; Chethan et al., 2020; Venkatramanan et al., 2021). The burning of rice residue results in almost 100% loss of C and N, while 20–60% loss of P, K, and S immersed in the residue (Porichha et al., 2021).

The three main principles of conservation agriculture (CA) (minimum soil disturbance, crop diversification, and permanent soil cover) contribute to protecting the soil from erosion and degradation, improving soil quality and biodiversity, preserving the natural resources, and increasing their use efficiency, optimizing crop yields, while imparting environmental sustainability (Sharma et al., 2022a). Conservation tillage (CT), such as no-tillage or reduced tillage, decreases soil disturbance, protects the soil against erosion, and increases soil organic matter (Busari et al., 2015). Recent studies have also shown that CT protects the life cycle of arthropods, increases their diversity (Rivers et al., 2016), and plays a major role in shaping microbial communities (Morugán-Coronado et al., 2022). Similarly, zero tillage with crop residue management effectively improves soil organic matter (SOM) and maintains crop yields (Bhattacharyya et al., 2012; Singh et al., 2018; Das et al., 2021; Saurabh et al., 2021). Other management practices, such as straw mulching or incorporation, and tillage have significant effects on total SOM and soil enzymes (Song et al., 2016; Zhang et al., 2016; Akhtar et al., 2019; Qin et al., 2021).

Direct seeded rice (DSR) technology, one of the important components of CA, has evolved to overcome the problems associated with soil, water, and the environment under PTR (Kumar and Ladha, 2011). Under the DSR technique, three basic operations, namely, puddling, transplanting, and ponding of water, are omitted. Consequently, DSR helps in reducing the total water requirement in rice production approximately by 30% (Joshi et al., 2013). However, a yield penalty of 10% has been recorded in zero tilled DSR rice but the yield of succeeding zero tilled wheat increased by 21%, showing the net benefit of it in the rice–wheat system (Sharma et al., 2019).

The surface retention of rice residue by direct seeding the Rabi crop with zero till using innovative farm machineries, such as Zero-till Drill, Mulcher, Rotary Disc Drill, and Happy Seeder, are emerging as feasible options for rice residue management (Sidhu et al., 2015). Furthermore, the direct seeding of Rabi crop with these farm machineries consumes less fuel compared to the conventional tillage system (Pratibha et al., 2015). The *in-situ* surface retention of crop residues as full or anchored also boosts soil health by improving soil physical properties (Das et al., 2021; Saurabh et al., 2021), soil organic carbon, and labile carbon fractions (Liu et al., 2013; Gupta et al., 2022); increasing moisture retention; regulating temperature (Fu et al., 2021); enhancing nutrient availability (Moharana et al., 2012) and root absorption; suppressing weeds (Nikolić et al., 2021); decreasing salinity (Prajapat et al., 2020); encouraging soil biological activity (Singh et al., 2018); and controlling crop pests and diseases (El-Shater and Yigezu, 2021). Moreover, crop diversification reduces pressure in current agriculture and maintains or even enhances soil microbial abundance as crop diversification has shown benefits for soil macro- and microorganisms, while maintaining crop yields (Baldwin-Kordick et al., 2022; Morugán-Coronado et al., 2022; Rai et al., 2022).

Barley (*Hordeum vulgare* L.) is the world's fourth most essential cereal crop after wheat, rice, and maize produced in more than 100 countries (Giraldo et al., 2019). Barley can be successfully grown under adverse climatic conditions of drought, salinity, and alkalinity. It is usually used as food for human beings and feed for animals, and it is considered superior to wheat as it lacks gluten. Furthermore, barley is an industrial crop and the crop of interest for entrepreneurs, farmers, and researchers. The importance of barley as a functional food is mainly due to its potentialities in the production of healthy food, as an excellent source of dietary fiber, and having β-glucan.

The introduction of green gram (*Vigna radiata* L.) in the cereal–cereal cropping system is in use for ages as it is beneficial in sustaining the productivity of the system in several ways. It maintains nitrogen balance in the agroecosystems and provides an opportunity for more grain and protein production. Mungbean-based wheat systems sustain productivity through moisture conservation, stable economic benefits, and improvement in soil nutrition and organic matter over time (Das et al., 2021; Saurabh et al., 2021).

Different tillage and residue management practices and diverse cropping systems have an effect on soil biological health, which include soil organic matter, microbial population, microbial biomass carbon (MBC), microbial biomass nitrogen, and microbial enzymatic activities (Johansen et al., 2012; Singh et al., 2018; Das et al., 2021). Whereas the soil biological indices are the important tools conveyed through several properties and denote the quality of soil in terms of sustainability (Lehman et al., 2015). These indices are dynamic soil properties that are very sensitive to land management, natural disturbances, and chemical contaminants (Herrick, 2000). In general, biological indicators that describe soil organisms mediated soil processes are the most informative about soil function (Paz-Ferreiro and Fu, 2016). In this context, it is important to understand which indicators to test, what information is needed for the appropriate management of soil, and where to get this information.

A soil biological index (SBI), which is an integrated expression of the most sensitive attributes, if found, can monitor soil health after barley in a holistic way for making the most sustainable management choices. To fill this gap, a systematic study was conducted to (1) evaluate the combined effect of cropping systems, tillage, and residue retention on crop yield; (2) identify key biological soil health indicators; and (3) develop a soil quality index to identify the best practices for the rice–barley cropping system in IGP of India.

## Materials and methods

### Experimental site and soil

The study was carried out at the Research Farm of ICAR-Indian Institute of Wheat and Barley Research, Karnal (29°42'12”N, 76°59'36”E and 245 m msl), from 2013–2014 to 2017–2018. The soil of the experimental farm is sandy loam in texture with 62.2% sand, 26.9% silt, and 10.9% clay. The soil is normal in electrical conductivity (EC_1:2_ 0.24 dS/m), alkaline in reaction (pH_1:2_ 8.3), low in oxidizable organic carbon (0.42), low in KMnO_4_-N (177.0 kg/ha), and medium in Olsen's–P (18.4 kg/ha) and NH_4_OAc–K (222.3 kg/ha). The climate of the region is semi-arid and sub-tropical with hot-dry summer (April to June), hot-humid rainy season (July to September), and cool-dry winter (October to March). The average annual maximum and minimum air temperatures are 29.9°C and 17.1°C, respectively. The average annual rainfall of the region is 670 mm, out of which 75–80% is received during the southwest monsoon (July to September) period. The meteorological parameters recorded during the experimental period are depicted in Supplementary Figure 1.

### Experimental setup and treatments

The experiment was initiated in the rainy season of 2013 with nine treatments of tillage and residue management in the rice–barley and rice–barley–green gram cropping systems in randomized block design with three replications. The treatments were T1: Reduced till direct seeded rice–zero till barley (RTDSR–ZTB); T2: Reduced till direct seeded rice–zero till barley–green gram (RTDSR–ZTB–Gg); T3: Zero till direct seeded rice–zero till barley–zero till green gram (ZTDSR–ZTB–ZTGg); T4: Reduced till direct seeded rice–zero till barley + rice residue at 4 t/ha (RTDSR–ZTB_RR4_); T5: Reduced till direct seeded rice–zero till barley + rice residue at 6 t/ha (RTDSR–ZTB_RR6_); T6: Un-puddled transplanted rice–zero till barley–green gram (UPTR–ZTB–Gg); T7: Un-puddled transplanted rice–zero till barley + rice residue 4 t/ha (UPTR–ZTB_RR4_); T8: Un-puddled transplanted rice–zero till barley + rice residue 6 t/ha (UPTR–ZTB_RR6_); and T9: Puddled transplanted rice–reduced till barley (PTR–RTB). The gross plot size was 4 m × 10 m under each treatment. The details of the crop rotation, tillage, and residue management are summarized in Table 1.

**Table 1:** Detailed description of tillage and residue management practices in rice–barley cropping system.

**Treatments depiction**	**Crop rotations (Rainy–winter–summer)**	**Tillage and crop establishment method**	**Residue management**	**Fertilizer doses (N+ P_2_O_5_, kg ha^−1^) and application**	**Irrigation management**
T1: RTDSR–ZTB	Rice–barley	Reduced tillage direct seeded rice (RTDSR)–zero tilled barley (ZTB)	Anchored rice residue retained on soil surface and all barley residue were removed	Rice: 60 + 30; P_2_O_5_ as basal at the time of sowing through DAP. N in three equal splits at sowing; at 21–25 and at 42–45 days after sowing (DAS). Barley: 90 + 40 Full P_2_O_5_ and half N as basal. Remaining half N as top dressing after first irrigation through urea	**Rice:** Soil was kept wet for the first 20 days followed by irrigation at an interval of 10–12 days depending on the field conditions. **Barley**: Three irrigations at critical crop growth stages (CRI, panicle emergence and grain formation)
T2: RTDSR–ZTB–Gg	Rice–barley–green gram	Reduced tillage direct seeded rice (RTDSR)–zero tilled barley (ZTB)–conventional tillage green gram (Gg)	Anchored rice residue retained on soil surface, barley residue removed and full green gram residue incorporated	Rice: 60 + 30; P_2_O_5_ as basal at the time of sowing through DAP. N in three equal splits at sowing; at 21–25 and at 42–45 days after sowing (DAS). Barley: 90 + 40; Full P_2_O_5_ and half N as basal. Remaining half N as top dressing after first irrigation through urea. Green gram: 20 + 40; applied as basal.	**Rice**: Soil was kept wet for the first 20 days followed by irrigation at an interval of 10–12 days depending on the field condition. **Barley**: Three irrigations at critical crop growth stages. **Green gram:** First irrigation at 20 DAS and thereafter two irrigations as per need.
T3: ZTDSR–ZTB–ZTGg	Rice–barley–green gram	Zero tillage DSR (ZTDSR)– zero tilled barley (ZTB)–zero tillage green gram (ZTGg)	Anchored residue of all the three crops retained on soil surface	Rice: 60 + 30; P_2_O_5_ as basal at the time of sowing through DAP. N in three equal splits at sowing; at 21–25 and at 42–45 days after sowing (DAS). Barley: 90 + 40; Full P_2_O_5_ and half N as basal. Remaining half N as top dressing after first irrigation through urea. Green gram: 20 + 40; applied as basal.	**Rice:** Soil was kept wet for the first 20 days followed by irrigation at an interval of 10-12 days depending on the field condition. **Barley:** Three irrigations at critical crop growth stages. **Green gram**: First irrigation at 20 DAS and two more irrigation as per need
T4: RTDSR–ZTB_RR4_	Rice–barley	Reduced tillage direct seeded rice (RTDSR)–zero tilled barley (ZTB)	Rice residue @ 4 t ha^−1^ retained on soil surface (RR4) and all barley residue were removed	Rice: 60 + 30; P_2_O_5_ as basal at the time of sowing through DAP. N in three equal splits at sowing; at 21–25 and at 42–45 days after sowing (DAS). Barley: 90 + 40; Full P_2_O_5_ and half N as basal. Remaining half N as top dressing after first irrigation through urea.	**Rice:** Soil was kept wet for the first 20 days followed by irrigation at an interval of 10–12 days depending on the field conditions. **Barley:** Three irrigations at critical crop growth stages.
T5: RTDSR–ZTB_RR6_	Rice–barley	Reduced tillage direct seeded rice (RTDSR)–zero tilled barley (ZTB)	Rice residue @ 6 tha^−1^ retained on soil surface (RR6) and all barley residue were removed	Rice: 60 + 30; P_2_O_5_ as basal at the time of sowing through DAP. N in three equal splits at sowing, at 21–25 and at 42–45 days after sowing (DAS). **Barley:** 90 + 40; Full P_2_O_5_ and half N as basal. Remaining half N as top dressing after first irrigation through urea	**Rice:** Soil was kept wet for the first 20 days followed by irrigation at an interval of 10–12 days depending on the field condition. **Barley:** Three irrigations at critical crop growth stages.
T6: UPTR–ZTB–Gg	Rice–barley–green gram	Unpuddled transplanted rice (UPTPR)–zero tillage barley (ZTB)–conventional tillage green gram (Gg)	Anchored rice residue retained on soil surface, barley residue removed and full green gram residue incorporated	Rice: 60 + 30; P_2_O_5_ as basal at the time of transplanting through DAP. N in three equal splits at 7–10 days after transplanting (DAT), at 21–25 and at 42–45 DAT through urea Barley: 90 + 40; Full P_2_O_5_ and half N as basal. Remaining half N as top dressing after first irrigation through urea. Green gram: 20 + 40; applied as basal.	**Rice:** Ponding of water (2–3 cm) for one month followed by light irrigations to keep soil moist depending on the field condition. **Barley:** Three irrigations at critical crop growth stages. **Green gram:** First irrigation 20 at DAS and two more irrigations as per need.
T7: UPTR–ZTB_RR4_	Rice–barley	Unpuddled transplanted rice (UPTPR)–zero tillage barley (ZTB)	Rice residue @ 4 t ha^−1^ retained on soil surface (RR4) and all barley residue were removed	Rice: 60 + 30; P_2_O_5_ as basal at the time of transplanting through DAP. N in three equal splits at 7–10 days after transplanting (DAT); at 21–25 and at 42–45 DAT through urea. Barley: 90 + 40; Full P_2_O_5_ and half N as basal. Remaining half N as top dressing after first irrigation through urea.	**Rice**: Ponding of water (2–3 cm) for 1 month followed by light irrigations to keep soil moist depending on the field condition. **Barley**: Three irrigations at critical crop growth stages.
T8: UPTR–ZTB_RR6_	Rice–barley	Unpuddled transplanted rice (UPTPR)–zero tillage barley (ZTB)	Rice residue @ 6 t ha^−1^ retained on soil surface (RR6)and all barley residue were removed	Rice: 60 + 30; P_2_O_5_ as basal at the time of transplanting through DAP. N in three equal splits at 7–10 days after transplanting (DAT); at 21–25 and at 42–45 DAT through urea Barley: 90 + 40; Full P_2_O_5_ and half N as basal. Remaining half N as top dressing after first irrigation through urea.	**Rice:** Ponding of water (2-3 cm) in plots for one month followed by light irrigations to keep soil moist depending on the field conditions. **Barley**: Three irrigations at critical crop growth stages.
T9: PTR–RTB	Rice–barley	Puddled transplanted rice (PTPR)–reduced tillage barley (RTB)	All residue removed	Rice: 60 + 30; P_2_O_5_ as basal at the time of transplanting through DAP. N in three equal splits at 7–10 days after transplanting (DAT); at 21–25 and at 42–45 DAT through urea. Barley: 90 + 40; Full P_2_O_5_ and half N as basal. Remaining half N as top dressing after first irrigation through urea.	**Rice:** Continuous flooding of 5 cm depth for 1 month followed by light irrigation at hair line crack in soil. **Barley:** Three irrigations at critical crop growth stages.

At the beginning of the experiment (May–June 2013), the field was plowed and leveled with a laser land leveler. The first crop of rice was grown as per treatments; however, the zero and minimum tillage treatments could not be applied in the first rice crop as the soil was plowed and prepared uniformly to begin the experiment. For subsequent rice crops, two passes of harrows were made followed by planking, and direct seeding was performed with a multi-crop seed drill fitted with inclined seed distribution plates in the reduced till treatments. The seed rate for DSR was 20 kg/ha. For ZTDSR, rice was directly sown in green gram residues in the month of June by using a multi-crop turbo happy seeder. For both the transplanted rice treatments (UPTR and PTR), the nursery was raised in separate plots and transplanted at the age of 30 days in the plots prepared as per tillage. The sowing in the nursery was done on the date of the sowing of DSR. For UPTR, the field was plowed with 2 passing of harrows followed by planking. The plots were ponded with water, and direct transplanting was performed manually without puddling. Under PTR, the field was harrowed two times after harvesting the barley crop. At the time of transplanting, puddling was done with a rotavator in ponded water conditions, and seedlings were transplanted manually in the puddled field. ZT barley and green gram were sown with a turbo happy seeder using 100 and 25 kg/ha seed rates, respectively. For RT barley, two harrowing and planking were done after rice harvesting in respective plots followed by the sowing of barley with a seed drill. Green gram was knocked down with the spray of 2,4-D at the flowering stage for sowing of succeeding zero-till rice. In RT and UPTR treatments, the green gram was incorporated into the soil at the flowering stage. All the crops were sown/transplanted at 20 cm row spacing. The cultivars used were Basmati CSR30 of rice, DURB52 of barley, and SML668 of green gram.

For controlling the germinated weeds in ZT plots, glyphosate 41% SL at 900 ml a.i./ha was sprayed 7–10 days before the sowing of crops. In DSR and ZT barley plots, pendimethalin 30% EC at 1,000 ml a.i./ha was sprayed just after sowing to control the first flush of weeds. In DSR, post-emergence application of bispyribac sodium 10% SC at 25 ml a.i./ha was done at 20–25 days after sowing (DAS) followed by 2,4-D amine 58% at 500 ml a.i./ha at 30 DAS to control growing weeds. In un-puddled and transplanted rice plots, pre-emergence application of pretilachlor 50% EC at 500 ml a.i./ha followed by post-emergence application of bispyribac sodium 10% SC was done to control the weeds. In barley, post-emergence spray of 2,4-D amine 58% SL at 500 ml a.i./ha and pinoxaden 5% EC at 50 ml a.i./ha was done after the first irrigation. Other standard recommended practices of the region were followed for insect-pest management. At maturity, rice and barley crops were harvested excluding the two border rows. The biomass was sun-dried and threshed to separate the grains. Per plot grain yield of both crops was expressed as grain yield in t/ha.

### Soil sampling and storage

Soil samples were collected at the end of the fifth cropping cycle, after the harvesting of barley crops during 2017–18. Samples were (0–15 cm depth) collected with the metal auger (5 cm diameter) near the root zone after the removal of the crop litter and stubble present. Three subsamples were taken from each plot to form one composite sample per plot. The soil was transported to the laboratory immediately after sampling and stored at 4°C until the analyses were conducted.

### Soil biological properties

Soil microbial biomass carbon (MBC) was determined using the fumigation-extraction method by fumigating 15 g of soil with ethanol-free chloroform followed by 0.5 M K_2_SO_4_ extraction (w:v 1:4); additionally, 15 g of soil was extracted with K_2_SO_4_ without fumigation. MBC flush was calculated using the relationship: MBC = [(1/0.38) × C-flush] (Vance et al., 1987). Soil respiration (SR0) was determined by measuring released CO_2_ (Stotzky, 2016). This assay was carried out in glass bottles (300 ml) and plastic cups containing soil with 5 ml of sodium hydroxide (1 mol/L). The C-CO_2_ collected in the alkaline solution was determined by titration of the residual NaOH with chloric acid (0.25 mol/L) after the addition of 2.5 ml of BaCl_2_.2H_2_O (1 mol/L) and phenolphthalein indicator. The C-CO_2_ produced was expressed in mg CO_2_-C/g soil week. Soil respiration (SR) was also performed after amendment with glucose (100 mg glucose/kg dry soil). The soil enzyme alkaline phosphatase (AP, pH 11.0) (EC 3.1.3.1) was measured by estimating the concentration of p-nitrophenol released on incubation of soil with p-nitrophenyl phosphate, used as substrate, and was expressed as μmol p-nitrophenol (p-NP) produced/g dry weight of soil h (Tabatabai and Bremner, 1969). The nitrate reductase activity (NR; EC 1.7.1.1) of soil was estimated in 0.19 M ammonium chloride buffer (pH 8.5) using 25 mM KNO_3_ as substrate (Han et al., 2013). The peroxidase (PO; EC 1.11.1.7) enzymes in soil were estimated colorimetrically as oxidation of L-3,4-dihydroxyphenylalanine in the presence of hydrogen peroxide (Sinsabaugh, 2010). Total microbial activity (TMA) in soil was determined by fluorescein diacetate (FDA) hydrolysis by mixing soil with 60 mM potassium phosphate buffer (pH 7.6) and 3′ 6′-diacetylfluorescein. The concentration of fluorescein released during the assay was measured spectrophotometerically at 490 nm and expressed as μg fluorescein g/soil h (Green et al., 2006). Ergosterol (EG) estimation in soil was done by HPLC using acetonitrile and methanol as eluting reagents with UV detection at 282 nm (Young, 1995). The extraction of ergosterol was carried out by treating the soil with methanol and 2 M sodium hydroxide in a culture tube and heating it for 50 s in a domestic microwave (2,450 MHz and 750 W). Total glomalin-related soil proteins (T-GRSP) estimation was carried out in the soil of all the treatments. The estimation of T-GRSP was carried out and expressed as μg/g dry weight of soil (Wright and Upadhyaya, 1996); 1 g of the air-dried soil was added to 8 ml sodium citrate (20 mM, pH 7.0) and was autoclaved at 121°C for 1 h, and then centrifuged at 5,000 rpm for 20 min. The concentration of T-GRSP was determined by Bradford assay with bovine serum albumin as the standard and was expressed as μg/g dry weight of soil.

### Microbial population

Microbial communities in the rhizosphere soils were determined by examining microbial populations by serial dilution method (Chandra et al., 2020). For bacterial (BA) population count, nutrient agar (Himedia^®^) was used. Potato dextrose agar (Himedia^®^) was used for the fungal count, while actinobacterial isolation agar (Himedia^®^) was used for the enumeration of actinobacteria (AC) populations. The collected soils of each treatment were serially diluted, and 1 ml of soil suspension each from 10^−5^ to 10^−8^ dilutions was spread on the respective media-filled plate in triplicate. Petri plates were then incubated at 28 ± 2°C. Bacterial colonies appearing within 48 h were counted, while fungal and actinobacterial colonies were counted after 3–4 and 6–7 days, respectively. Results were expressed in colony-forming units (CFUs)/g of soil sample.

### Computation of soil biological index

The principal component analysis (PCA) technique (Bastida et al., 2006; Barman et al., 2017) using SPSS (version 16.0) was used to identify the minimum dataset based on different soil biological parameters analyzed. Through this mathematical procedure, a (smaller) number of uncorrelated variables (PC) were transformed from several (possibly) correlated variables. The sensitive indicators of SBI were selected based on the score of the factor in PCA analysis carried out with determined parameters (FDA, MBC, SR, NR, and FN). This data reduction analysis provides four principle components; the first component explained the highest variance in the results. Five sensitive parameters with the highest weight from the four principle components of PCA were selected for the development of SBI. The final value of SBI was normalized (0 to 1 scale) because the absolute values of some parameters were lower than other parameters. In this study, the sensitive parameters, namely, FDA, MBC, and FN, functioned on “the more the better,” i.e., the more fungi population, the better nutrient availability for the barley crop. In the case of sensitive parameters, SR and NR functioned as “the less the better.” For normalization, Eq. 1 was used which followed the sigmoidal curve, between 0–1. SBI consisted of summing these four sensitive parameters but to bring the value between 0 and 1, the final value of SBI was normalized as follows:


(1) $$\begin{array}{l} \text{Y =}\frac{\text{a}}{\text{1+}{\left(\frac{\text{x}}{{\text{x}}_{\text{0}}}\right)}^{\text{b}}} \end{array}$$

where ‘a' is the maximum value, in this case, a = 1; “x” is the value of the parameter in question in each case, which is the unknown; “x_0_” is the mean value of each sensitive parameter of the rice–barley system in the study; and “b” is the slope of the equation. We used optimized values of “b” that fit the sigmoid curve tending to 1 for 5 sensitive parameters. The value of curves ranged between 0 and 1.

The sensitive variables for each observation were weighted by using the PCA results. Each PC explained a certain amount (%) of the variation in the total dataset. This percentage, divided by the total percentage of variation explained by all PCs with eigen vectors >1, provided the weighted factor for variables chosen under a given PC (Table 2). The weighting of each variable in the principle component was multiplied with each normalized value and then summed up using the following equation:


(2) $$\begin{array}{l} \text{SBI}=\overset{n}{\underset{i=1}{\sum }}w_{i}s_{i} \end{array}$$

where S = indicator score (Equation. 1) and W = the weighing factor obtained from PCA.

**Table 2:** Performance of soil biological indicators in terms of factor loading/eigen vector values in principal component analysis.

**PCs**	**PC1**	**PC2**	**PC3**	**PC4**
Eigen value	5.6	1.7	1.3	1.1
Per cent variance	46.4	14.0	10.8	8.8
Cumulative percentage	46.4	60.4	71.2	80.0
**Factor loading/eigen vector**				
FDA	**0.91**	0.16	0.11	0.12
MBC	**0.81**	0.23	0.32	0.27
AP	**0.86**	0.33	0.21	0.19
T-GRSP	0.68	0.19	0.58	0.29
EG	0.15	0.36	0.29	0.58
NR	0.03	0.00	**0.89**	−0.01
PO	0.32	0.85	0.27	0.26
SR	0.08	**0.96**	−0.02	−0.05
SR0	0.44	0.43	0.65	−0.18
BA	0.25	0.10	−0.15	0.70
AC	−0.77	0.03	0.09	−0.11
FN	0.10	−0.11	0.01	**0.82**

MBC, microbial biomass carbon; SR0, soil respiration without glucose; SR, soil respiration with glucose AP, alkaline phosphatase; NR, nitrate reductase; PO, peroxides; FDA, fluorescein diacetate hydrolysis; EG, Ergosterol; T-GRSP, glomalin; BA, Bacteria; AC, Actinobacteria; FN, Fungi. Bold values indicate the eigen vectors within 10% of the highest factor loadings.

Higher index scores were assumed to mean better soil biological quality or greater performance of soil function. The SBI estimated from the above method was validated against the system yield of rice–barley cropping system by computing multiple regression coefficients.

### Sustainable yield index

To express the overall impact of treatments on productivity, the sustainable yield index (SYI) was calculated based on the barley grain equivalent yield (BEY) of the rice–barley cropping system of 5 years. The sustainable yield index (SYI) was computed using the following equation:


(3) $$\begin{array}{l} \text{SYI }=\text{ i}-\sigma /{\text{Y}}_{\text{Max}} \end{array}$$

where i is the mean BEY of the respective treatment, σ is the standard deviation of the treatment, and Y_Max_ is the maximum BEY of any treatment in the experiment in any year.

### System productivity

System productivity of the rice–barley system in terms of barley equivalent yield (BEY t/ha) was calculated by taking into account the grain yields and market prices of rice and barley crops as follows:


$$\begin{array}{l} \text{BEY (t/ha) }=\;\frac{rice\;grain\;yield\;(t/ha)\;\times \;price\;of\;rice\;grain\;}{price\;of\;barley\;grain\;}\\ \;+barley\;grain\;yield\;(t/ha) \end{array}$$

### Statistical analysis

The statistical analysis of data was carried out using the ANOVA technique for randomized block design in the SAS program. The standard error of the mean with respect to each parameter was calculated. For comparisons where significant F probabilities (*p* < 0.05) were found, we used the DUNCAN's Multiple Range Test (DMRT). Data were tested for normality and transformed suitably if not following a normal distribution. PCA and correlation matrix were carried out in the R computer software. MS Excel was used for normalization models and graphs.

## Results

### Crop yield and system productivity

**Figure 1:**
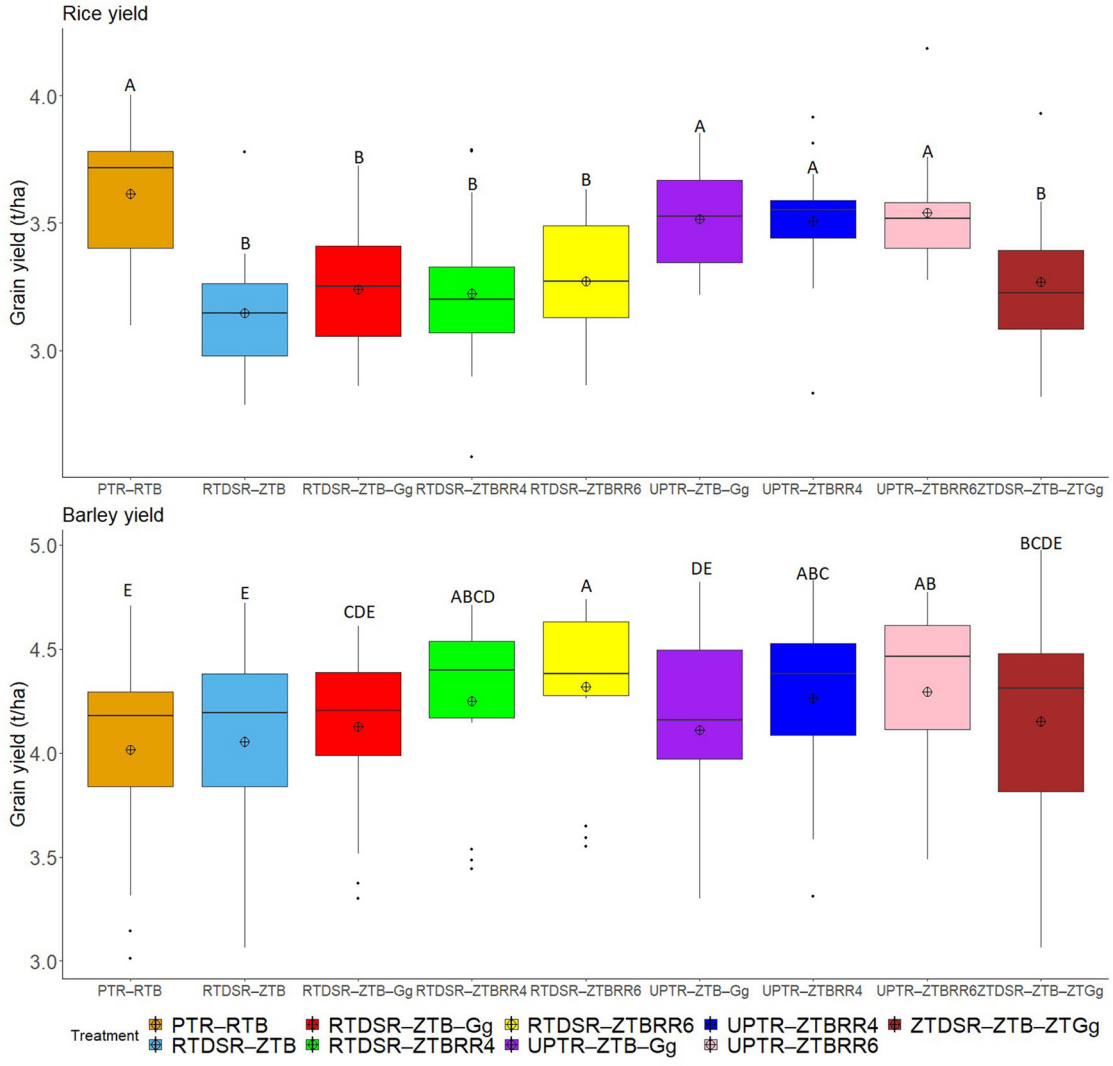
Effect of tillage and residue management practices on rice and barley grain yield. RTDSR–ZTB, reduced till direct seeded rice–zero till barley; RTDSR–ZTB–Gg, reduced till direct seeded rice–zero till barley–green gram; ZTDSR–ZTB–ZTGg, zero till direct seeded rice–zero till barley–zero till green gram; RTDSR–ZTBRR4, reduced till–direct seeded rice–zero till barley + rice residue at 4 t/ha; RTDSR–ZTBRR6, reduced till direct seeded rice–zero till barley + rice residue at 6 t/ha; UPTR–ZTB–Gg, un-puddled transplanted rice–zero till barley–green gram; UPTR–ZTBRR4, un-puddled transplanted rice–zero till barley + rice residue 4 t/ha; UPTR–ZTBRR6, un-puddled transplanted rice–zero till barley + rice residue 6 t/ha; PTR–RTB, puddled transplanted rice–reduced till barley; R, residue. Box plots with different capital letters are significantly different (*p* < 0.05) using DUNCAN's multiple range test.

**Figure 2:**
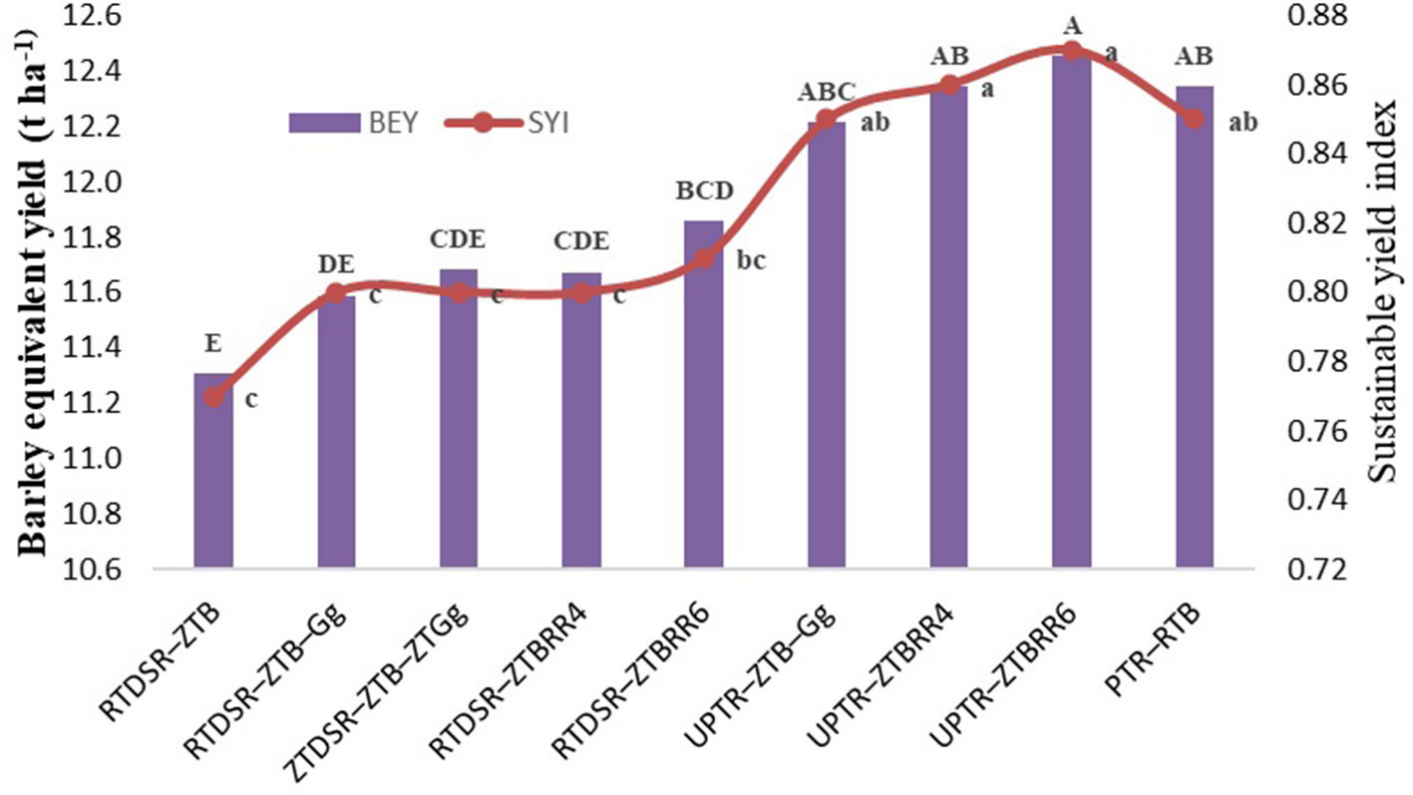
Effect of tillage and residue management practices on system productivity and SYI. RTDSR–ZTB, reduced till direct seeded rice–zero till barley; RTDSR–ZTB–Gg, reduced till direct seeded rice–zero till barley–green gram; ZTDSR–ZTB–ZTGg, zero till direct seeded rice–zero till barley–zero till green gram; RTDSR–ZTB_RR4_, reduced till–direct seeded rice–zero till barley + rice residue at 4 t/ha; RTDSR–ZTB_RR6_, reduced till direct seeded rice–zero till barley + rice residue at 6 t/ha; UPTR–ZTB–Gg, un-puddled transplanted rice–zero till barley–green gram; UPTR–ZTB_RR4_, un-puddled transplanted rice–zero till barley + rice residue 4 t/ha; UPTR–ZTB_RR6_, un-puddled transplanted rice–zero till barley + rice residue 6 t/ha; PTR–RTB, puddled transplanted rice-reduced till barley; R, residue. Bars with different capital letters for BEY and small letters for SYI are significantly different (*p* < 0.05) using DUNCAN's multiple range test.

Different tillage and residue management practices significantly affected the yield of rice and barley (Figure 1) and system productivity as barley equivalent yield (Figure 2). On the pooled basis, the conventional rice followed by reduced till barley (PTR–RTB) maintained the highest rice grain yield (3.61 t/ha). There was no significant reduction in grain yield of rice grown without puddling irrespective of tillage and residue management practices. The rice grain yield was significantly lower under all the treatments of reduced and zero tillage adopted in rice. Moreover, the mean grain yield of rice was 10.6% lower under DSR than that of PTR.

The RTB after PTR recorded the lowest grain yield of barley crop (4.02 t/ha). Zero tilled barley without residue incorporation also recorded poor grain yield (4.05 t/ha). The mungbean residue incorporation or rice residue retention under ZT significantly improved the barley grain yield and RTDSR–ZTB_RR6_ recorded the highest barley grain yield (4.32 t/ha), followed by UPTR–ZTB_RR6_ (4.29 t/ha) and UPTR–ZTB_RR4_ (4.29 t/ha). The rice residue retention under RTDSR–ZTB_RR6_ enhanced the grain yield of barley by 6.7% over RTDSR–ZTB.

The system productivity calculated in terms of barley equivalent yield showed that tillage and residue management treatment involving UPTR–ZTB_RR6_ proclaimed the highest BEY (12.45 t/ha), followed by UPTR–ZTB_RR6_ (12.35 t/ha), PTR–RTB (12.35 t/ha), and UPTR–ZTB–Gg (12.21 t/ha).

### Sustainable yield index

The sustainable yield index of the rice–barley cropping system is depicted in Figure 2. The SYI followed the trends of system productivity, wherein UPTR–ZTB_RR6_ recorded the highest SYI, followed by the UPTR–ZTB_RR4_ and UPTR–ZTB–Gg. Among the conservation tillage, RTDSR—ZTB_RR6_ was recorded with the highest SYI.

### MBC and soil respiration

Soil MBC was found to be significantly (*p* < 0.05) affected by different nutrient management practices. Significantly (*p* < 0.05) highest MBC was observed in the UPTR–ZTB_RR6_ (518.78 mg/kg soil), followed by UPTR–ZTB_RR4_ (488.43 mg/kg soil) (Table 3). Similarly, soil respiration (SR0) also significantly (*p* < 0.05) varied in different treatments. Glucose-induced soil respiration (SR) was the highest in UPTR–ZTB_RR6_ (77.78 mg CO_2_-C/g soil week), followed by UPTR–ZTB_RR4_ (75.86 mg CO_2_-C/g soil week); however, SR0 was significantly (*p* < 0.05) higher in UPTR–ZTB_RR4_ (70.69 mg CO_2_-C/g soil week) and PTR–RTB (70.35 mg CO_2_-C/g soil week) compared with other management practices (Table 3).

**Table 3:** Effect of tillage and residue management practices on soil biological properties.

**Treatments**	**MBC**	**SR**	**SR0**	**AP**	**NR**	**PO**	**FDA**	**EG**	**T-GRSP**
RTDSR–ZTB	106.4^h^	74.8^e^	44.6^g^	39.7^e^	640.1^g^	6.7^c^	1.0^ed^	14.6^abc^	17.4^g^
RTDSR–ZTB–Gg	214.0^f^	75.5^c^	46.0^f^	47.1^d^	967.3^f^	7.1^c^	1.1^ed^	9.03^c^	14.3^h^
ZTDSR–ZTB–ZTGg	264.1^e^	75.6^c^	54.9^e^	42.0^e^	1011^f^	9.1^c^	2.1^cd^	12.8^bc^	23.7^f^
RTDSR–ZTB_RR4_	398.3^c^	73.8^g^	62.1^c^	54.9^c^	1225.0^e^	12.0^b^	3.0^c^	19.1^a^	44.8^b^
RTDSR–ZTB_RR6_	398.7^c^	74.6^f^	57.6^d^	71.7^b^	1752.8^c^	11.4^b^	5.2^b^	17.6^ab^	38.4^c^
UPTR–ZTB–Gg	299.1^d^	71.0^h^	55^e^	48.4^e^	2816.3^a^	11.2^b^	1.1^de^	19.7^bc^	35.3^d^
UPTR–ZTB_RR4_	488.4^b^	75.8^b^	70.6^a^	73.5^b^	1560.4^d^	13.0^b^	4.56^b^	12.1^bc^	43.8^b^
UPTR–ZTB_RR6_	518.7^a^	77.7^a^	65.7^b^	82.1^a^	2145.4^b^	15.5^a^	6.4^a^	20.3^a^	49.2^a^
PTR–RTB	156.2^g^	75.1^d^	70.3^a^	41.8^e^	2777.3^a^	7.0^c^	0.8^e^	8.7^c^	26.6^e^
**Contrast**									
RB vs. RBG	0.0061	<0.0001	<0.0001	<0.0001	0.0058	0.0023	0.8291	0.2596	<0.0001
RTDSR vs. ZTDSR	0.2181	<0.0001	<0.0001	<0.0001	0.0194	0.7235	0.0036	0.2399	<0.0001
RTDSR vs. UPTR	<0.0001	<0.0001	<0.0001	<0.0001	<0.0001	<0.0001	<0.0001	0.2824	<0.0001
UTPR vs. PTR	<0.0001	0.0014	<0.0001	<0.0001	<0.0001	<0.0001	<0.0001	0.0015	<0.0001
Mulch vs. anchored R	<0.0001	<0.0001	<0.0001	<0.0001	<0.0001	<0.0001	0.0037	0.0271	<0.0001

FDA (μg fluorescein g soil^−1^ h^−1^), MBC (mg kg^−1^ soil), AP (μmol p-nitrophenol g^−1^ h^−1^), T-GRSP (μg kg^−1^), EG (μg g^−1^ soil), NR (μg ml^−1^ hr^−1^), PO (units μg^−1^), SR (mg CO_2_-C g^−1^ soil week^−1^), SR0 (mg CO_2_-C g^−1^ soil week^−1^). RTDSR–ZTB, reduced till direct seeded rice–zero till barley; RTDSR–ZTB-Gg, reduced till direct seeded rice–zero till barley–green gram; ZTDSR–ZTB–ZTGg, zero till direct seeded rice–zero till barley–zero till green gram; RTDSR–ZTB_RR4_, reduced till–direct seeded rice–zero till barley + rice residue at 4 t ha^−1^; RTDSR–ZTB_RR6_, reduced till direct seeded rice–zero till barley + rice residue at 6 t ha^−1^; UPTR–ZTB-Gg, un-puddled transplanted rice–zero till barley–green gram; UPTR–ZTB_RR4_, un-puddled transplanted rice–zero till barley + rice residue 4 t ha^−1^; UPTR-ZTB_RR6_, un-puddled transplanted rice–zero till barley + rice residue 6 t ha^−1^; PTR–RTB, puddled transplanted rice–reduced till barley; R, residue. Means with different small letters within column are significantly different (*p* < 0.05) using DUNCAN's Multiple Range Test.

### Microbial enzymes

In this study, values of AP varied significantly (*p* < 0.05) with different tillage and residue incorporation practices. The activity of AP was significantly (*p* < 0.05) high in UPTR–ZTB_RR6_ (82.19 μmol p-nitrophenol/g h), followed by UPTR–ZTB_RR4_ (73.5 μmol p-nitrophenol/g h) and PTR–RTB (71.69 μmol p-nitrophenol/g h) (Table 3). The NR activity was significantly (*p* < 0.05) high in UPTR–ZTB–Gg (2,816.32 μg/ml h) and PTR–RTB (2,777.35 μg/ml h) (Table 3). The residue-amended treatment UPTR–ZTB_RR6_ (15.49 units/μg) demonstrated significantly (*p* < 0.05) high PO activity (Table 3).

### Microbial indicators

In this study, the FDA was significantly (*p* < 0.05) high in UPTR–ZTB_RR6_ (6.48 μg fluorescein g/soil h), followed by UPTR–ZTB_RR4_ (5.21 μg fluorescein g/soil h), and RTDSR–ZTB_RR4_ (4.51 μg fluorescein g/soil h) (Table 3). EG content was high in residue-amended plots as it was significantly (*p* < 0.05) high in UPTR–ZTB_RR6_ (20.30 μg/g soil) (Table 3). T-GRSP in the soil was significantly (*p* < 0.05) high in treatment UPTR–ZTB_RR6_ (49.22 μg/kg), followed by UPTR–ZTB_RR4_ (44.23 μg/kg) (Table 3).

### Microbial population

The significantly (*p* < 0.05) high bacterial population was in treatment UPTR–ZTB_RR6_ (93.3 × 10^5^ CFU/g), while the population of Actinobacteria was significantly (*p* < 0.05) high in ZTDSR–ZTB–ZTGg (54.67 × 10^4^ CFU/g) (Table 4). The significantly (*p* < 0.05) high fungal population was recorded in treatment UPTR–ZTB_RR6_ (47.0 × 10^3^ CFU/g) and UPTR–ZTB_RR4_ (44.0 × 10^3^ CFU/g) (Table 4).

**Table 4:** Effect of tillage and residue management practices on soil microbial populations.

**Treatments**	**BA**	**AC**	**FN**
RTDSR–ZTB	93^a^	50^ab^	42.3^bc^
RTDSR–ZTB–Gg	82^abcd^	46^abc^	41.3^bc^
ZTDSR–ZTB–ZTGg	78^d^	54.6^a^	41.3^bc^
RTDSR–ZTB_RR4_	81^bcd^	48.6^ab^	41.3^bc^
RTDSR–ZTB_RR6_	81.3^bcd^	46.6^abc^	39.3^c^
UPTR–ZTB–Gg	90.3^abc^	51.33^ab^	38^c^
UPTR–ZTB_RR4_	92.3^ab^	43^bc^	44^b^
UPTR–ZTB_RR6_	93.3^a^	38.6^c^	47^a^
PTR–RTB	79^cd^	48^ab^	34.3^d^
**Contrast**			
RB vs. RBG	0.2131	0.0241	0.2453
RTDSR vs. ZTDSR	0.122	0.0406	0.931
RTDSR vs. UPTR	0.0133	0.1142	0.0156
UTPR vs. PTR	0.0059	0.264	0.0009
Mulch vs. anchored R	0.6927	0.0053	0.8551

BA, Bacteria (CFU g^−1^ × 10^5^); AC, Actinobacteria (CFU g^−1^ × 10^4^); FN, Fungi (CFU g^−1^ × 10^3^). RTDSR–ZTB, reduced till direct seeded rice–zero till barley; RTDSR–ZTB–Gg, reduced till direct seeded rice–zero till barley–green gram; ZTDSR–ZTB–ZTGg, zero till direct seeded rice–zero till barley–zero till green gram; RTDSR–ZTB_RR4_, reduced till–direct seeded rice–zero till barley + rice residue at 4 t ha^−1^; RTDSR–ZTB_RR6_, reduced till direct seeded rice–zero till barley + rice residue at 6 t ha^−1^; UPTR–ZTB–Gg, un-puddled transplanted rice–zero till barley–green gram; UPTR–ZTB_RR4_, un-puddled transplanted rice–zero till barley + rice residue 4 t ha^−1^; UPTR–ZTB_RR6_, un-puddled transplanted rice–zero till barley + rice residue 6 t ha^−1^; PTR–RTB, puddled transplanted rice–reduced till barley; R, residue. Means with different small letters within same column are significantly different (*p* < 0.05) using DUNCAN's Multiple Range Test.

### Principal component analysis and soil biological index

**Figure 3:**
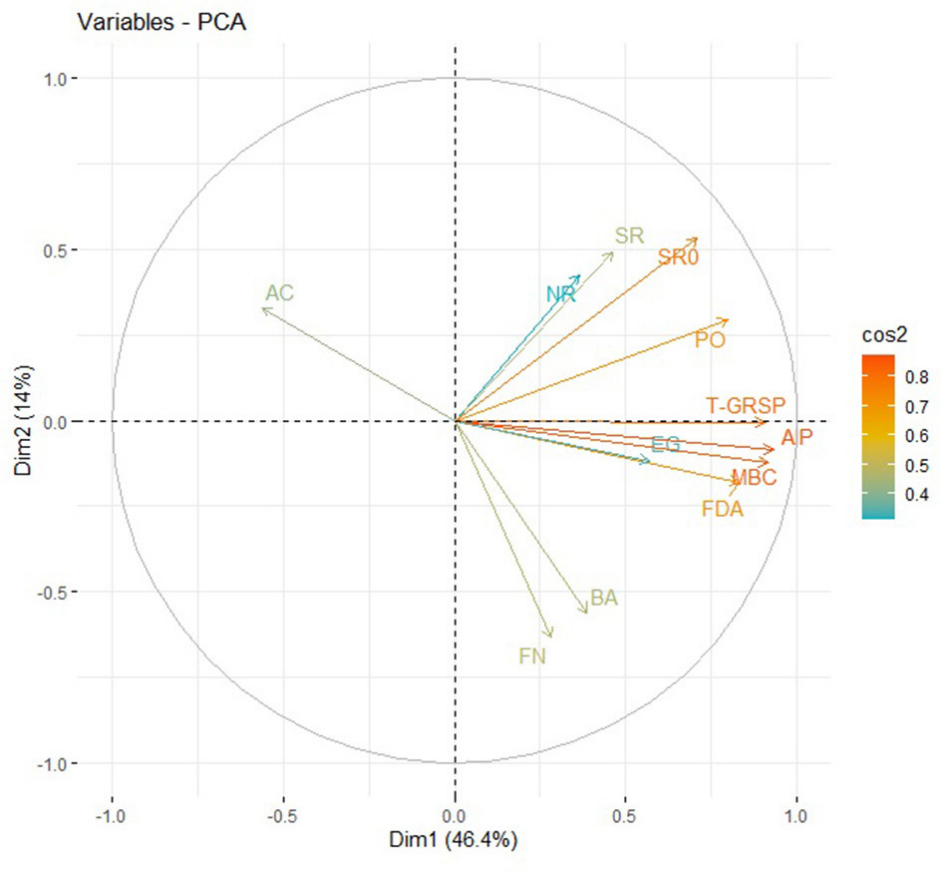
Graphic display biplot for soil biological attributes as influenced by tillage and residue management practices. BA, Bacteria; AC, Actinobacteria; FN, Fungi; NR, Nitrate reductase activity; SR0, Soil respiration; PO, Peroxidase; SR, Glucose-induced soil respiration; EG, Ergosterol; T-GSRP, Total glomalin-related soil proteins; FDA, Fluorescein diacetate hydrolysis; MBC, Microbial biomass carbon; AP, Alkaline phosphatase.

**Figure 4:**
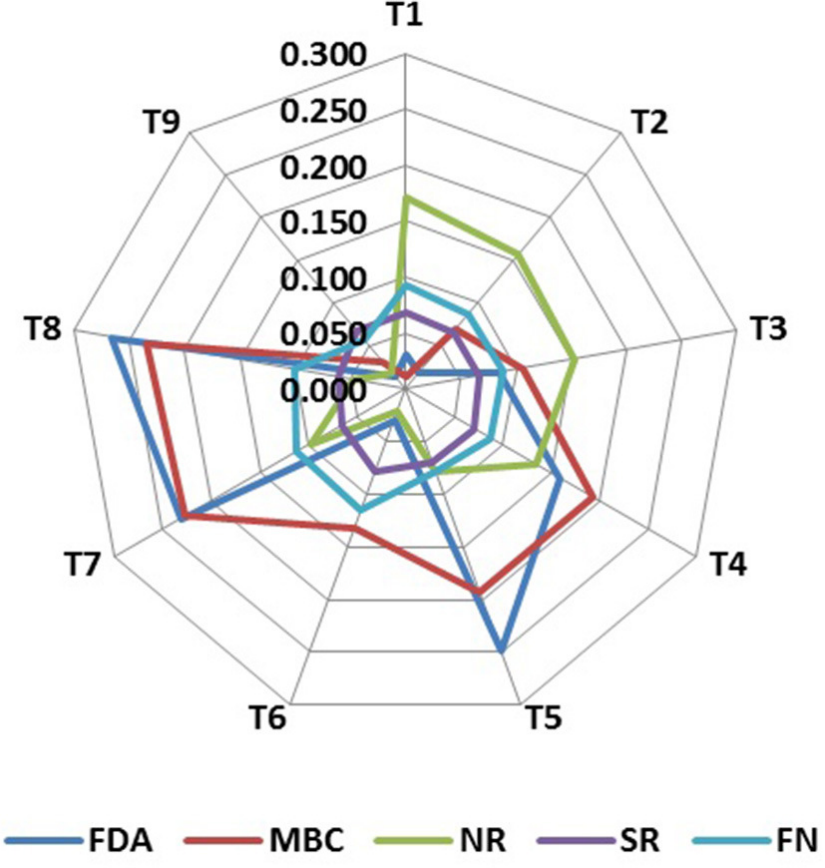
Radar graph depicting the average linear scores of key indicators as influenced by soil-nutrient management treatments. T1: reduced till direct seeded rice–zero till barley (RTDSR–ZTB); T2: reduced till direct seeded rice–zero till barley–green gram (RTDSR–ZTB–Gg); T3: zero till direct seeded rice–zero till barley–zero till green gram (ZTDSR–ZTB–ZTGg); T4: reduced till direct seeded rice–zero till barley + rice residue at 4 t/ha (RTDSR–ZTB_RR4_); T5: reduced till direct seeded rice–zero till barley + rice residue at 6 t/ha (RTDSR–ZTB_RR6_); T6: un-puddled transplanted rice–zero till barley–green gram (UPTR–ZTB–Gg); T7: un-puddled transplanted rice–zero till barley + rice residue 4 t/ha (UPTR–ZTB_RR4_); T8: un-puddled transplanted rice–zero till barley + rice residue 6 t/ha (UPTR–ZTB_RR6_); T9: puddled transplanted rice–reduced till barley (PTR–RTB). FDA, Fluorescein diacetate hydrolysis; MBC, Microbial biomass carbon; NR, Nitrate reductase activity; SR, Glucose-induced soil respiration; FN, Fungi.

Soil biological properties had a significant effect on the productivity of the rice–barley cropping system, which indicates their significance in the determination of SBI. Principal component analysis (PCA) with all the soil biological indicators showed that a variance of 80% was explained in the PCA of 12 variables, and 4 PCs were extracted with eigen values >1 (Table 2). FDA (fluorescein diacetate), MBC (microbial biomass carbon), and AP (alkaline phosphatase) have 46.4% of the total variance, which were highly weighted variables in PC1 (Table 2). To select the minimum variables for the development of soil biological index (SBI), out of the three variables of PC1, FDA and MBC were chosen as sensitive parameters in PC1. Among the two enzyme activity indicators (FDA and AP), AP was removed from the dataset as it has a high correlation (*p* = 0.90) with FDA, and FDA represents the overall soil enzyme activity (Supplementary Figure 2). PC2 has a 14.0% of the total variance, and SR (soil respiration with glucose) was selected to be the sensitive parameter. The third PC has 10.8% of the total variation, and NR (nitrate reductase) was considered a highly weighted eigenvector, while PC4 has 8.8% of the total variation, and FN (fungi) was selected as a sensitive parameter. The final sensitive biological indicators consisted of FDA, MBC, SR, NR, and FN (Figure 3). Based on the average linear scores of sensitive parameters, the FDA showed the highest scores followed by MBC, fungi, SR, and NR (Figure 4).

**Figure 5:**
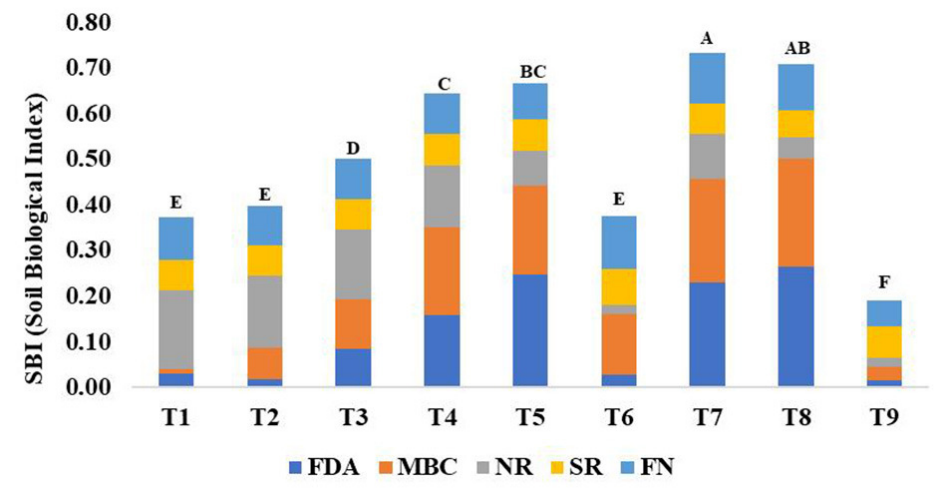
Effect of different tillage and residue management practices on soil biological index and the individual contribution of each of the key indicators. T1: reduced till direct seeded rice–zero till barley (RTDSR–ZTB), T2: reduced till direct seeded rice–zero till barley–green gram (RTDSR–ZTB–Gg), T3: zero till direct seeded rice–zero till barley–zero till green gram (ZTDSR–ZTB–ZTGg), T4: reduced till–direct seeded rice–zero till barley + rice residue at 4 t/ha (RTDSR–ZTB_RR4_), T5: reduced till direct seeded rice–zero till barley + rice residue at 6 t/ha (RTDSR–ZTB_RR6_), T6: un-puddled transplanted rice–zero till barley–green gram (UPTR–ZTB–Gg), T7: un-puddled transplanted rice–zero till barley + rice residue 4 t/ha (UPTR–ZTB_RR4_), T8: un-puddled transplanted rice–zero till barley + rice residue 6 t/ha (UPTR–ZTB_RR6_), T9: puddled transplanted rice–reduced till barley (PTR–RTB). Bars with different capital letters are significantly different (*p* < 0.05) using DUNCAN's multiple range test. FDA, Fluorescein diacetate hydrolysis; MBC, Microbial biomass Carbon; NR, Nitrate reductase activity; SR, Glucose-induced soil respiration; FN, Fungi.

**Figure 6:**
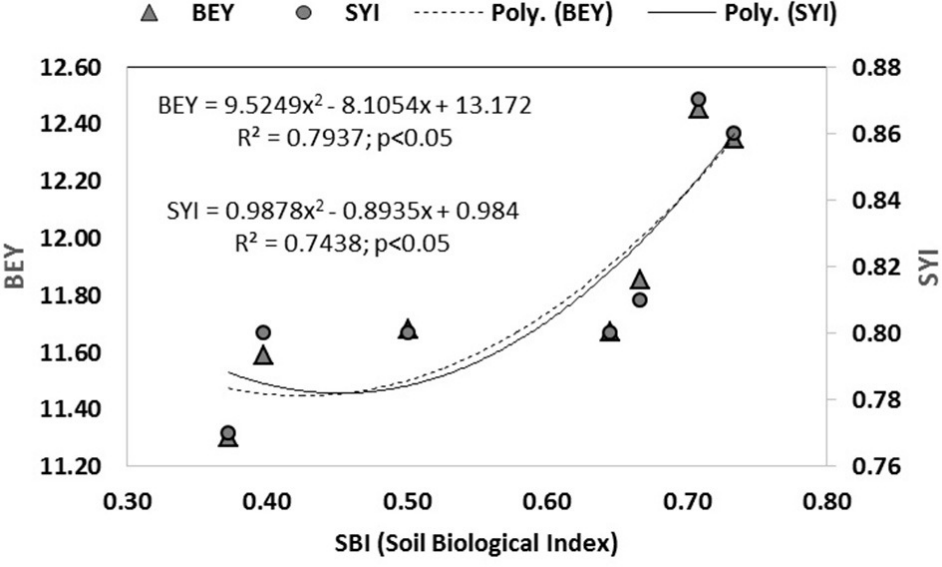
Relationship between soil biological index (SBI) with system productivity (BEY) and sustainable yield index (SYI) under tillage and residue management practices.

Based on these sensitive parameters, SBI was computed for different treatments used in the study. Treatments showed significant (*p* < 0.05) differences for SBI (Figure 5). The traditional practice of rice–barley (PTR–RTB) showed the minimum value of SBI. Rice residue retention improved the SBI under RTDSR and UPTR and had a greater impact than mungbean residues. UPTR–ZTB with rice residue retention of 4 and 6 t/ha and RTDSR–ZTB with rice residue retention of 6 t/ha recorded higher SBI values over other treatments. The regression relationship of SBI with BEY and SYI showed that there was an improvement in system productivity and sustainable yield index with an increase in SBI (Figure 6).

## Discussion

### Crop yields, system productivity, and sustainability

Conservation agriculture practices have been advocated for mitigating the ill effects of cereal-based intensive agriculture while preserving soil health with a substantial reduction in the cost of production and minimal environmental footprints (Johansen et al., 2012; Mishra et al., 2022). Different tillage and residue retention or incorporation practices in the rice–barley cropping system had a significant effect on the productivity and soil quality in the present experiment. The conventional practice of puddling and transplanting (puddled transplanted rice) continued to claim higher grain yield in our 5 years cropping cycle. Flooding of rice under puddled transplanting provides several congenial changes suitable for rice to grow faster and healthy. Some of these are suppression of weeds during early growth, availability of micronutrients (Fe and Zn), no infestation of nematodes, etc. (Balasubramanian and Hill, 2002; Kumar and Ladha, 2011; Ismail et al., 2012; Chaudhary et al., 2022). The lower yields in RTDSR and ZTDSR in our experiment are attributed to poor plant stand and infestation of weeds during early vegetative stages (data not presented). The transplanting of rice in un-puddled soil with reduced tillage (UPTR) gave a statistically equivalent yield to that of puddled transplanted rice without adversely affecting the yield of succeeding crop of barley with rice or mungbean residues. Therefore, the un-puddled transplanting of rice with two shallow tillage and direct rice transplanting in shallow water (2–3 cm depth) may be considered as minimum tillage when compared to conventional puddled rice, which involves deep tillage in a dry field, followed by repeated wet tillage for puddling (Haque et al., 2016).

The barley grain yield was significantly affected by the tillage practice of previous rice and residue treatments (Figure 1). The adverse effect of puddling in rice is obvious in barley grain yield, which was recorded as the lowest among all the practices. A number of adverse effects of puddling on the soil after rice such as destruction of soil structure and dispersion of clay, which creates a layer impermeable for root penetration, formation of sub-soil hardpan due to continuous tillage in wet soil, water logging in winter crop in the event of rains, etc., have been reported for lower yield of succeeding winter season crop (Kirchhof et al., 2000; Haque et al., 2016; Kalita et al., 2020; Kumar et al., 2023). The sowing of barley involving zero tillage (ZTB) without residue retention or mungbean residue incorporation was also found to be a poor performer in terms of barley grain yield. Barley yield was higher after RTDSR and ZTDSR practices than that of PTR and UPTR. The better soil conditions in reduced and zero tillage practices might have resulted in a higher yield of the subsequent barley crop. Furthermore, the green gram residue incorporation or rice residue retention substantially improved the grain yield of barley, showing a beneficial effect of conservation agriculture practices. Our results are in accordance with the earlier findings showing the additive effect of residue retention on yields of wheat under zero tillage (Yadvinder-Singh et al., 2004; Jat et al., 2014; Magar et al., 2022). The higher rice yield and comparable barley yield under UPTR–ZTB_RR6_ resulted in overall higher BEY *viś-a-viś* SYI of the rice–barley cropping system.

### Soil biological parameters

The MBC is one of the most labile of the carbon pools comprising organic matter, which varies with different management practices (Zhang et al., 2021; Cordeiro et al., 2022). Nutrient availability potential represents an increase in MBC than an increase in total organic matter (Sharma et al., 2022b). In this study, residue incorporation enhanced the value of MBC, which aligns with other reports that state that organic sources like farmyard manure, vermicompost, and crop residues decompose slowly and result in the accumulation of organic carbon in soil (Singh et al., 2015, 2018). Several studies have also reported that tillage and crop rotation influence MBC (Zuber et al., 2018; Malobane et al., 2020; Nyambo et al., 2021; Saurabh et al., 2021). In this study, higher MBC in residue-retained treatments may also be due to that residue incorporation modifies microbial distribution in soil which encourages the proliferation of microbial population, supporting higher MBC (Li et al., 2018; Meena R.S et al., 2020). It is also reported that organic amendments stimulate microorganisms to produce enzymes related to the nitrogen and phosphorus cycles and soil organic carbon accumulation to boost soil microorganisms and enzyme activities (Rashid et al., 2016; Jacoby et al., 2017).

SR represents the value of CO_2_ released from the soil due to the decomposition of organic matter present in the soil by microbes and the respiration of plant roots and other soil fauna. Soil respiration is very much sensitive to excessive tillage and incorporation of crop residues (Bhowmik et al., 2017; Usero et al., 2021). In this study, SR was also found to vary significantly (*p* < 0.05) in different treatments and was significantly (*p* < 0.05) high in zero till or reduced till plots. Likewise, treatments with residue retention also had high SR, which may be due to the enhancement of soil moisture because of residue retention (Kallenbach et al., 2019) and also due to the conversion of organic matter into available form of nutrients such as phosphate as PO_4_, nitrate-nitrogen as NO_3_, and sulfate (Fu et al., 2021).

Microbial enzymes represent soil quality as they facilitate diverse functions and are the most sensitive parameters, which change frequently with the physical and chemical properties of the soil (Burns et al., 2013). As reported earlier, fertilization and crop management practices significantly change soil microbial enzymes (Singh et al., 2015; Singh and Sharma, 2020). Crop residue-amended soils were better suited for microbial development, which may have enhanced nutrient mobilization and prevented the soil from fixing the available P (Sharma et al., 2022c). The fluctuations in enzyme activity could easily indicate the changes in the biological dynamics of the soil. Phosphatases in the soil serve important roles in the soil system and are an excellent indication of soil fertility by driving the mineralization of Po to accessible Pi (Saha et al., 2022). As a result of the long-term crop residue management techniques, the soil's microbial population and biomass C or N significantly increased, giving energy and an ideal environment for the development of soil enzymes (Xomphoutheb et al., 2020). In this study, alkaline phosphatase enzymes were found high in residue-incorporated treatments, which is in accordance with earlier studies (Gupta et al., 2022; Sharma et al., 2022a). However, NR is the enzyme that catalyzes the reduction of ${\text{NO}}_{3}^{-}$ to ${\text{NO}}_{2}^{-}$ in soil and also represents nitrogen immobilization (Canfield et al., 2010; Grzyb et al., 2021). In this study, the activity of NR enzymes was the highest in the treatment incorporated with green gram, which harbors rhizobium in the root nodules that convert atmospheric N_2_ to NO${}_{3}^{-}$. Hence, the presence of ${\text{NO}}_{3}^{-}$ in the soil induces the NR activity of the treatment (Abbasifar et al., 2020). Peroxidase activity was the highest in the treatment incorporated with rice residue of 6 t/ha. Rice straw consists of lignocellulosic material that mainly consists of cellulose (24.0%), hemicelluloses (27.8%), and lignin (13.5%) (Chen et al., 2020). PO enzymes predominantly involve in carbon mineralization processes including humification and lignin degradation (Kumar and Chandra, 2020), and the presence of such a high concentration of lignocellulosic material in soil activates PO enzymes.

Repeated puddling adversely affects soil physical properties by dismantling soil aggregates, reducing permeability in subsurface layers, and forming hardpans at shallow depths. Hence, significantly (*p* < 0.05) higher microbial populations are found in un-puddled plots. Un-puddled conditions were therefore congenial for the proliferation of microbial population in soils, which may be directly linked with the maintenance of aggregates of soil particles (Bhowmik et al., 2017). The population of actinomycetes was the highest in green gram grown in crop rotation as it is a leguminous crop, and its rhizosphere harbors several types of microbes. Their roots secrete exudates encompassing easily available compounds, such as amino acids and carbohydrates, which can also stimulate the microbial population and their activity (Badri and Vivanco, 2009). It is possible that the stronger plant-root association also increased the microbial population, resulting in the use of more root exudate secreted by the plant (Rai et al., 2021; Chandra et al., 2022b). In no-till treatment, fungal biomass is higher than in tilled soils due to less breakage of hyphal networks and less damage to mycorrhizal associations (Schalamuk et al., 2004; Wilkes et al., 2021). The symbiotic association between microbes and roots is generally measured by ergosterol content as it is also called a biomarker for fungi. EG is mainly found in the fungal cell membrane, which controls the activity of membrane-bound enzymes and their permeability (Jordá and Puig, 2020). Similarly, T-GRSP is another biomarker for mycorrhizal associations with roots and is a glycoprotein produced by the hyphae of mycorrhiza, and it is mainly responsible for the formation of soil aggregates (Singh et al., 2015), which were found higher in the un-puddled treatment in the study. FDA hydrolysis enzyme activity corresponds to total microbial activity (Chandra et al., 2022a). FDA hydrolysis enzyme activity was also found to be positively correlated with MBC and AP (Supplementary Figure 2). As microbial population and soil enzymes were high in ZT, un-puddled and rice residue-incorporated treatments; hence, FDA activity was also the highest in these treatments.

### Soil biological index

The principal component analysis indicated five sensitive biological parameters such as FDA, MBC, SR, NR, and FN (Figure 3). Among these, FDA and MBC had higher weightage in SBI (Figures 4, 5). FDA related to the total soil microbial activity can be considered an important indicator of soil microbial activity (Chandra et al., 2022a). MBC acts as a sink of soil nutrients and an important indicator of soil quality and biological activity (Srivastava et al., 2020). MBC is also indicative of soil organic matter changes due to soil management practices (Lal, 2020). There was significant variation in SBI under different tillage and residue management practices in the study. The highest SBI was found in the treatments with rice residue retention (Figure 5). These treatments increased the microbial population in the soil, which leads to an increase in the soil biological quality that can be monitored by the assessment of these five sensitive parameters. Agronomic management practices have a greater influence on soil microbial activities (Chen et al., 2021). Enhanced soil microbial activity and microbial populations under residues retained plots have led to improved SBI (Das et al., 2021). Un-puddled transplanted rice followed by zero till barley along with retention of rice residues improved the soil biological quality over the period of 5 years. The SBI values were validated against barley equivalent yield, and the sustainable yield index elaborated that higher SBI was associated with better system productivity and sustainability of rice–barley cropping system (Figure 6).

## Conclusion

The adoption of reduced or no tillage in the short term reduced the grain yield of basmati rice more than that of puddled transplanting. The barley yield under zero tillage improved significantly with the incorporation of green gram residue or retention of 4–6 t/ha rice residue. Based on the results of the study, the partial conservation agriculture practice of un-puddled transplanted rice with reduced tillage followed by zero tilled barley and retention of 4–6 t/ha rice residues (UPTR–ZTB _RR6_ or UPTR–ZTB_RR4_) provided the highest system productivity (12.45 t/ha and 12.35 t/ha), sustainable yield index (0.87 and 0.86), and improved soil biological health as evidenced by the highest soil biological index (0.71 and 0.73). For farmers using rice residues for other purposes, green gram residue incorporation in the UTPR–ZTB may be adopted as it also provided statistically similar system productivity as that of UPTR–ZTB_RR6_. This study is a step forward toward a better understanding of the impact of tillage and residue management practices on soil biological and biochemical properties under the rice–barley cropping system, and long-term studies are needed for providing more reliable and predictable indicators to monitor the sustainability of semi-arid soils.

## Data availability statement

The original contributions presented in the study are included in the article/Supplementary material, further inquiries can be directed to the corresponding authors.

## Author contributions

Conceptualization: PC and AK. Methodology: PC and GeS. Formal analysis: AB, KP, and AR. Writing—original draft preparation: PC and KP. Writing—review and editing: AK, OA, RV, and KK. Editing and supervision: GyS. All authors have read and agreed to the published version of the manuscript.

## References

[B1] Abbasifar A. ValizadehKaji B. Iravani M. A. (2020). Effect of green synthesized molybdenum nanoparticles on nitrate accumulation and nitrate reductase activity in spinach. J. Plant Nutr. 43, 13–27. 10.1080/01904167.2019.1659340

[B2] Akhtar K. Wang W. Ren G. Khan A. Feng Y. Yang G.. (2019). Integrated use of straw mulch with nitrogen fertilizer improves soil functionality and soybean production. Environ. Int. 132, 105092. 10.1016/j.envint.2019.105092 31442740

[B3] Ambast S. K. Tyagi N. K. Raul S. K. (2006). Management of declining groundwater in the Trans Indo-Gangetic Plain (India): Some options. Agric. Water Manag. 82, 279–296. 10.1016/j.agwat.2005.06.005

[B4] Badri D. V. Vivanco J. M. (2009). Regulation and function of root exudates. Plant. Cell Environ. 32, 666–681. 10.1111/j.1365-3040.2009.01926.x 19143988

[B5] Balasubramanian V. Hill J. (2002). “Direct seeding of rice in Asia: Emerging issues and strategic research needs for the 21st century,” in Direct Seeding: Research Strategies and Opportunities 15–39.

[B6] Baldwin-Kordick R. De M. Lopez M. D. Liebman M. Lauter N. Marino J.. (2022). Comprehensive impacts of diversified cropping on soil health and sustainability. Agroecol. Sustain. Food Syst. 46, 331–363. 10.1080/21683565.2021.2019167

[B7] Barman A. Pandey R. N. Singh B. Das B. (2017). Manganese deficiency in wheat genotypes: Physiological responses and manganese deficiency tolerance index. J. Plant Nutr. 40, 2691–2708. 10.1080/01904167.2017.1381717

[B8] Bastida F. Moreno J. Hernández T. Garcia C. (2006). Microbiological degradation index of soils in a semiarid climate. Soil Biol. Biochem. 38, 3463–3473. 10.1016/j.soilbio.2006.06.001

[B9] Bhattacharyya R. Tuti M. D. Kundu S. Bisht J. K. Bhatt J. C. (2012). Conservation tillage impacts on soil aggregation and carbon pools in a sandy clay loam soil of the Indian Himalayas. Soil Sci. Soc. Am. J. 76, 617–627. 10.2136/sssaj2011.0320

[B10] Bhowmik A. Cloutier M. Ball E. Bruns M. A. (2017). Underexplored microbial metabolisms for enhanced nutrient recycling in agricultural soils. AIMS Microbiol. 3, 826–845. 10.3934/microbiol.2017.4.826 31294192 PMC6604955

[B11] Burns R. G. DeForest J. L. Marxsen J. Sinsabaugh R. L. Stromberger M. E. Wallenstein M. D.. (2013). Soil enzymes in a changing environment: Current knowledge and future directions. Soil Biol. Biochem. 58, 216–234. 10.1016/j.soilbio.2012.11.009

[B12] Busari M. A. Kukal S. S. Kaur A. Bhatt R. Dulazi A. A. (2015). Conservation tillage impacts on soil, crop and the environment. Int. Soil Water Conserv. Res. 3, 119–129. 10.1016/j.iswcr.2015.05.002

[B13] Canfield D. E. Glazer A. N. Falkowski P. G. (2010). The evolution and future of Earth's nitrogen cycle. Science 330, 192–196. 10.1126/science.1186120 20929768

[B14] Chandra P. Dhuli P. Verma P. Singh A. Choudhary M. Prajapat K.. (2020). Culturable microbial diversity in the rhizosphere of different biotypes under variable salinity. Trop. Ecol. 61, 291–300. 10.1007/s42965-020-00089-3

[B15] Chandra P. Gill S. C. Prajapat K. Barman A. Chhokar R. S. Tripathi S. C.. (2022a). Response of wheat cultivars to organic and inorganic nutrition: effect on the yield and soil biological properties. Sustainability 14, 9578. 10.3390/su14159578

[B16] Chandra P. Singh A. Prajapat K. Rai A. K. Yadav R. K. (2022b). Native arbuscular mycorrhizal fungi improve growth, biomass yield, and phosphorus nutrition of sorghum in saline and sodic soils of the semi–arid region. Environ. Exp. Bot. 201, 104982. 10.1016/j.envexpbot.2022.104982

[B17] Chaudhary A. Venkatramanan V. Kumar Mishra A. Sharma S. (2022). Agronomic and environmental determinants of direct seeded rice in South Asia. Circ. Econ. Sustain. 10.1007/s43615-022-00173-x 35573660 PMC9075927

[B18] Chen C. Chen Z. Chen J. Huang J. Li H. Sun S.. (2020). Profiling of chemical and structural composition of lignocellulosic biomasses in tetraploid rice straw. Polymers (Basel). 12, 340. 10.3390/polym12020340 32033358 PMC7077374

[B19] Chen Y.-P. Tsai C.-F. Rekha P. D. Ghate S. D. Huang H.-Y. Hsu Y.-H.. (2021). Agricultural management practices influence the soil enzyme activity and bacterial community structure in tea plantations. Bot. Stud. 62, 8. 10.1186/s40529-021-00314-9 34003387 PMC8131499

[B20] Chethan C. R. Singh P. K. Dubey R. P. Chander S. Gosh D. Choudhary V. K.. (2020). Crop residue management to reduce GHG emissions and weed infestation in Central India through mechanized farm operations. Carbon Manag. 11, 565–576. 10.1080/17583004.2020.1835387

[B21] Cordeiro C. F. dos S. Rodrigues D. R. Silva G. F. da Echer F. R. Calonego J. C. (2022). Soil organic carbon stock is improved by cover crops in a tropical sandy soil. Agron. J. 114, 1546–1556. 10.1002/agj2.21019

[B22] Das S. Bhattacharyya R. Das T. K. Sharma A. R. Dwivedi B. Meena M.. (2021). Soil quality indices in a conservation agriculture based rice-mustard cropping system in North-western Indo-Gangetic Plains. Soil Tillage Res. 208, 104914. 10.1016/j.still.2020.104914

[B23] El-Shater T. Yigezu Y. A. (2021). Can retention of crop residues on the field be justified on socioeconomic grounds? A case study from the mixed crop-livestock production systems of the moroccan drylands. Agronomy 11, 1465. 10.3390/agronomy11081465

[B24] Fu B. Chen L. Huang H. Qu P. Wei Z. (2021). Impacts of crop residues on soil health: a review. Environ. Pollut. Bioavailab. 33, 164–173. 10.1080/26395940.2021.1948354

[B25] Giraldo P. Benavente E. Manzano-Agugliaro F. Gimenez E. (2019). Worldwide research trends on wheat and barley: a bibliometric comparative analysis. Agronomy 9, 352. 10.3390/agronomy9070352

[B26] Green V. Stott D. Diack M. (2006). Assay for fluorescein diacetate hydrolytic activity: Optimization for soil samples. Soil Biol. Biochem. 38, 693–701. 10.1016/j.soilbio.2005.06.020

[B27] Grzyb A. Wolna-Maruwka A. Niewiadomska A. (2021). The significance of microbial transformation of nitrogen compounds in the light of integrated crop management. Agronomy 11, 1415. 10.3390/agronomy11071415

[B28] Gupta R. K. Hans H. Kalia A. Kang J. S. Kaur J. Sraw P. K.. (2022). Long-term impact of different straw management practices on carbon fractions and biological properties under riceandndash;wheat system. Agriculture 12, 1733. 10.3390/agriculture12101733

[B29] Han J.-G. Dou L. Zhu Y. Li P. (2013). Estimates of potential nitrate reductase activity in sediments: Comparisons of two incubation methods and four inhibitors. Environ. Earth Sci. 71, 419–425. 10.1007/s12665-013-2449-1

[B30] Haque M. E. Bell R. W. Islam M. A. Rahman M. A. (2016). Minimum tillage unpuddled transplanting: An alternative crop establishment strategy for rice in conservation agriculture cropping systems. F. Crop. Res. 185, 31–39. 10.1016/j.fcr.2015.10.018

[B31] Herrick J. (2000). Soil quality: An indicator of sustainable land management? Appl. Soil Ecol. 15, 75–83. 10.1016/S0929-1393(00)00073-1

[B32] Ismail A. M. Johnson D. E. Ella E. S. Vergara G. V. Baltazar A. M. (2012). Adaptation to flooding during emergence and seedling growth in rice and weeds, and implications for crop establishment. AoB Plants 2012, pls019. 10.1093/aobpla/pls019 22957137 PMC3434364

[B33] Jacoby R. Peukert M. Succurro A. Koprivova A. Kopriva S. (2017). The role of soil microorganisms in plant mineral nutrition-current knowledge and future directions. Front. Plant Sci. 8, 1617. 10.3389/fpls.2017.01617 28974956 PMC5610682

[B34] Jain N. Bhatia A. Pathak H. (2014). Emission of air pollutants from crop residue burning in India. Aerosol Air Qual. Res. 14, 422–430. 10.4209/aaqr.2013.01.003133971471

[B35] Jat R. K. Sapkota T. B. Singh R. G. Jat M. L. Kumar M. Gupta R. K. (2014). Seven years of conservation agriculture in a rice–wheat rotation of Eastern Gangetic Plains of South Asia: Yield trends and economic profitability. F. Crop. Res. 164, 199–210. 10.1016/j.fcr.2014.04.015

[B36] Johansen C. Haque M. E. Bell R. W. Thierfelder C. Esdaile R. J. (2012). Conservation agriculture for small holder rainfed farming: Opportunities and constraints of new mechanized seeding systems. F. Crop. Res. 132, 18–32. 10.1016/j.fcr.2011.11.026

[B37] Jordá T. Puig S. (2020). Regulation of ergosterol biosynthesis in saccharomyces cerevisiae. Genes (Basel). 11, 795. 10.3390/genes11070795 32679672 PMC7397035

[B38] Joshi E. Kumar D. Lal D. B. Nepalia V. Gautam P. Vyas A. K. (2013). Management of direct seeded rice for enhanced resource - use efficiency. Plant Knowl. J. 2, 119–134.36436645

[B39] Kakraliya S. K. Jat H. S. Sapkota T. B. Singh I. Kakraliya M. Gora M. K.. (2021). Effect of Climate-Smart Agriculture Practices on Climate Change Adaptation, Greenhouse Gas Mitigation and Economic Efficiency of Rice-Wheat System in India. Agric. 11, 1269. 10.3390/agriculture11121269

[B40] Kalita J. Ahmed P. Baruah N. (2020). Puddling and its effect on soil physical properties and growth of rice and post rice crops: A review. J. Pharmacog. Phytochem. 9, 503–510.

[B41] Kallenbach C. M. Conant R. T. Calderón F. Wallenstein M. D. (2019). A novel soil amendment for enhancing soil moisture retention and soil carbon in drought-prone soils. Geoderma 337, 256–265. 10.1016/j.geoderma.2018.09.027

[B42] Kirchhof G. Priyono S. Utomo W. Adisarwanto T. Dacanay E. V. So H. B. (2000). The effect of soil puddling on the soil, physical properties and the growth of rice and post-rice crops. Soil Tillage Res. 56, 37–50. 10.1016/S0167-1987(00)00121-5

[B43] Kumar A. Chandra R. (2020). Ligninolytic enzymes and its mechanisms for degradation of lignocellulosic waste in environment. Heliyon 6, e03170. 10.1016/j.heliyon.2020.e03170 32095645 PMC7033530

[B44] Kumar N. Chhokar R. S. Meena R. P. Kharub A. S. Gill S. C. Tripathi S. C.. (2021). Challenges and opportunities in productivity and sustainability of rice cultivation system: a critical review in Indian perspective. Cereal Res. Commun. 50, 573–601. 10.1007/s42976-021-00214-5 34642509 PMC8498983

[B45] Kumar P. Kumar A. Mittal S. (2004). Total factor productivity of crop sector in the indo-gangetic plain of India: Sustainability issues revisited. Indian Econ. Rev. 39, 169–201.

[B46] Kumar S. Gopinath K. A. Sheoran S. Meena R. S. Srinivasarao C. Bedwal S.. (2023). Pulse-based cropping systems for soil health restoration, resources conservation, and nutritional and environmental security in rainfed agroecosystems. Front. Microbiol. 13. 10.3389/fmicb.2022.1041124 36817102 PMC9935831

[B47] Kumar V. Ladha J. K. (2011). Direct seeding of rice: recent developments and future research needs. Adv. Agron. 111, 297–413. 10.1016/B978-0-12-387689-8.00001-136420033

[B48] Lal R. (2020). Soil organic matter and water retention. Agron. J. 112, 282. 10.1002/agj2.20282

[B49] Lehman R. M. Cambardella C. A. Stott D. E. Acosta-Martinez V. Manter D. K. Buyer J. S.. (2015). Understanding and enhancing soil biological health: the solution for reversing soil degradation. Sustainability 7, 988–1027. 10.3390/su7010988

[B50] Li L. Xu M. Eyakub Ali M. Zhang W. Duan Y. Li D. (2018). Factors affecting soil microbial biomass and functional diversity with the application of organic amendments in three contrasting cropland soils during a field experiment. PLoS ONE 13, e0203812. 10.1371/journal.pone.0203812 30212559 PMC6136761

[B51] Liu E. Yan C. Mei X. Zhang Y. Fan T. (2013). Long-term effect of manure and fertilizer on soil organic carbon pools in dryland farming in northwest china. PLoS ONE 8, e56536. 10.1371/journal.pone.0056536 23437161 PMC3577875

[B52] Magar S. T. Timsina J. Devkota K. P. Weili L. Rajbhandari N. (2022). Conservation agriculture for increasing productivity, profitability and water productivity in rice-wheat system of the Eastern Gangetic Plain. Environ. Challenges 7, 100468. 10.1016/j.envc.2022.100468

[B53] Malobane M. E. Nciizah A. D. Nyambo P. Mudau F. N. Wakindiki I. I. C. (2020). Microbial biomass carbon and enzyme activities as influenced by tillage, crop rotation and residue management in a sweet sorghum cropping system in marginal soils of South Africa. Heliyon 6, e05513. 10.1016/j.heliyon.2020.e05513 33294667 PMC7683310

[B54] Meena R. P. Venkatesh K. Khobra R. Tripathi S. C. Prajapat K. Sharma R. K.. (2020a). Effect of rice residue retention and foliar application of k on water productivity and profitability of wheat in North West India. Agron. 10, 434. 10.3390/agronomy10030434

[B55] Meena R. S. Kumar S. Datta R. Lal R. Vijayakumar V. Brtnicky M.. (2020b). Impact of Agrochemicals on Soil Microbiota and Management: A Review. Land 9, 34. 10.3390/land9020034

[B56] Mishra A. K. Shinjo H. Jat H. S. Jat M. L. Jat R. K. Funakawa S.. (2022). Farmers' perspectives as determinants for adoption of conservation agriculture practices in Indo-Gangetic Plains of India. Resour. Conserv. Recycl. Adv. 15, 200105. 10.1016/j.rcradv.2022.200105

[B57] Moharana P. Sharma B. M. Biswas D. Dwivedi B. Singh R. V. (2012). Long-term effect of nutrient management on soil fertility and soil organic carbon pools under a 6-year-old pearl millet-wheat cropping system in an Inceptisol of subtropical India. F. Crop. Res. 136, 32–41. 10.1016/j.fcr.2012.07.002

[B58] Morugán-Coronado A. Pérez-Rodríguez P. Insolia E. Soto-Gómez D. Fernández-Calviño D. Zornoza R. (2022). The impact of crop diversification, tillage and fertilization type on soil total microbial, fungal and bacterial abundance: A worldwide meta-analysis of agricultural sites. Agric. Ecosyst. Environ. 329, 107867. 10.1016/j.agee.2022.107867

[B59] Nikolić N. Loddo D. Masin R. (2021). Effect of crop residues on weed emergence. Agronomy 11, 163. 10.3390/agronomy11010163

[B60] Nyambo P. Thengeni B. Chiduza C. Araya T. (2021). Tillage, crop rotation, residue management and biochar influence on soil chemical and biological properties. South African J. Plant Soil 38, 390–397. 10.1080/02571862.2021.1962421

[B61] Pandey B. P. Kandel T. P. (2020). Response of rice to tillage, wheat residue and weed management in a rice-wheat cropping system. Agron. 10, 1734. 10.3390/agronomy10111734

[B62] Paz-Ferreiro J. Fu S. (2016). Biological indices for soil quality evaluation: perspectives and limitations. L. Degrad. Dev. 27, 14–25. 10.1002/ldr.2262

[B63] Porichha G. K. Hu Y. Rao K. T. Xu C. C. (2021). Crop residue management in india: stubble burning vs. other utilizations including bioenergy. Energies 14, 4281. 10.3390/en14144281

[B64] Prajapat K. Yadav R. Kumar A. Sharma C. (2020). Influence of sowing methods and mulching on soil salt dynamics and performance of fodder sorghum irrigated with saline water. J. Soil Salinity Water Quality. 10, 233–242.

[B65] Pratibha G. Srinivas I. Rao K. V. Raju B. M. K. Thyagaraj C. R. Korwar G. R.. (2015). Impact of conservation agriculture practices on energy use efficiency and global warming potential in rainfed pigeonpea–castor systems. Eur. J. Agron. 66, 30–40. 10.1016/j.eja.2015.02.001

[B66] Qin X. Huang T. Lu C. Dang P. Zhang M. Guan X.. (2021). Benefits and limitations of straw mulching and incorporation on maize yield, water use efficiency, and nitrogen use efficiency. Agric. Water Manag. 256, 107128. 10.1016/j.agwat.2021.107128

[B67] Rai A. K. Basak N. Soni P. G. Kumar S. Sundha P. Narjary B.. (2022). Bioenergy sorghum as balancing feedback loop for intensification of cropping system in salt-affected soils of the semi–arid region: Energetics, biomass quality and soil properties. Eur. J. Agron. 134, 126452. 10.1016/j.eja.2021.126452

[B68] Rai A. K. Dinkar A. Basak N. Dixit A. K. Das S. K. Dev I.. (2021). Phosphorus nutrition of oats genotypes in acidic soils: Exploiting responsive plant-microbe partnership. Appl. Soil Ecol. 167, 104094. 10.1016/j.apsoil.2021.104094

[B69] Rashid M. I. Mujawar L. H. Shahzad T. Almeelbi T. Ismail I. M. I. Oves M. (2016). Bacteria and fungi can contribute to nutrients bioavailability and aggregate formation in degraded soils. Microbiol. Res. 183, 26–41. 10.1016/j.micres.2015.11.007 26805616

[B70] Rivers A. Barbercheck M. Govaerts B. Verhulst N. (2016). Conservation agriculture affects arthropod community composition in a rainfed maize–wheat system in central Mexico. Appl. Soil Ecol. 100, 81. 10.1016/j.apsoil.2015.12.004

[B71] Saha R. Paswan A. Mazumdar S. P. Barman D. Majumdar B. Behera M. S.. (2022). Improvement in soil quality through tillage and residue management in Jute (Corchorus spp.) based cropping systems of Indo-Gangetic plains. Carbon Manag. 13, 205–215. 10.1080/17583004.2022.2068453

[B72] Saurabh K. Rao K. K. Mishra J. S. Kumar R. Poonia S. P. Samal S. K.. (2021). Influence of tillage based crop establishment and residue management practices on soil quality indices and yield sustainability in rice-wheat cropping system of Eastern Indo-Gangetic Plains. Soil Tillage Res. 206, 104841. 10.1016/j.still.2020.104841 33536693 PMC7722508

[B73] Schalamuk S. Velázquez S. Chidichimo H. Cabello M. (2004). Effect of no-till and conventional tillage on mycorrhizal colonization in spring wheat. Bol. la Soc. Argentina Bot. 39, 13–20.

[B74] Shahane A. A. Shivay Y. S. Prasanna R. Kumar D. (2020). Nutrient removal by rice–wheat cropping system as influenced by crop establishment techniques and fertilization options in conjunction with microbial inoculation. Sci. Rep. 10, 21944. 10.1038/s41598-020-78729-w 33319787 PMC7738681

[B75] Sharma P. C. Datta A. Yadav A. K. Choudhary M. Jat H. S. McDonald A. (2019). Effect of Crop Management Practices on Crop Growth, Productivity and Profitability of Rice–Wheat System in Western Indo-Gangetic Plains. Proc. Natl. Acad. Sci. India Sect. B Biol. Sci. 89, 715–727. 10.1007/s40011-018-0985-x

[B76] Sharma S. Kaur S. Parkash Choudhary O. Singh M. Al-Huqail A. A. Ali H. M.. (2022a). Tillage, green manure and residue retention improves aggregate-associated phosphorus fractions under rice-wheat cropping. Sci. Rep. 12, 7167. 10.1038/s41598-022-11106-x 35504974 PMC9064998

[B77] Sharma S. Singh P. Angmo P. Satpute S. (2022b). Total and labile pools of organic carbon in relation to soil biological properties under contrasting land-use systems in a dry mountainous region. Carbon Manag. 13, 352–371. 10.1080/17583004.2022.2089236

[B78] Sharma S. Vashisht B. B. Singh P. Singh Y. (2022c). Changes in soil aggregate-associated organic carbon, enzymatic activity, and biological pools under conservation agriculture based practices in rice–wheat system. Biomass Convers. Biorefinery. 10.1007/s13399-021-02144-y

[B79] Sidhu H. S. Singh M. Singh Y. Blackwell J. Lohan S. K. Humphreys E.. (2015). Development and evaluation of the Turbo Happy Seeder for sowing wheat into heavy rice residues in NW India. F. Crop. Res. 184, 201–212. 10.1016/j.fcr.2015.07.025

[B80] Singh G. Bhattacharyya R. Das T. K. Sharma A. Ghosh A. Das S.. (2018). Crop rotation and residue management effects on soil enzyme activities, glomalin and aggregate stability under zero tillage in the Indo-Gangetic Plains. Soil Tillage Res. 184, 291–300. 10.1016/j.still.2018.08.006

[B81] Singh G. Kumar D. Sharma P. (2015). Effect of organics, biofertilizers and crop residue application on soil microbial activity in rice – wheat and rice-wheat mungbean cropping systems in the Indo-Gangetic plains. Cogent Geosci. 1, 1085296. 10.1080/23312041.2015.1085296

[B82] Singh S. Sharma S. (2020). Temporal changes in rhizosphere biological soil quality indicators of wheat in response to nitrogen and straw incorporation. Trop. Ecol. 61, 328–344. 10.1007/s42965-020-00092-8

[B83] Sinsabaugh R. (2010). Phenol oxidase, peroxidase and organic matter dynamics of soil. Soil Biol. Biochem. 42, 391–404. 10.1016/j.soilbio.2009.10.01431841928

[B84] Song K. Yang J. Xue Y. Lv W. Zheng X. Pan J. (2016). Influence of tillage practices and straw incorporation on soil aggregates, organic carbon, and crop yields in a rice-wheat rotation system. Sci. Rep. 6, 36602. 10.1038/srep36602 27812038 PMC5095642

[B85] Srivastava P. Singh R. Bhadouria R. Tripathi S. Raghubanshi A. S. (2020). Temporal change in soil physicochemical, microbial, aggregate and available C characteristic in dry tropical ecosystem. CATENA 190, 104553. 10.1016/j.catena.2020.104553

[B86] Stotzky G. (2016). “Microbial Respiration,” in Methods of Soil Analysis Agronomy Monographs, ed. C. A. Black (Madison, WI: American Society of Agronomy) 1550–1572. 10.2134/agronmonogr9.2.c62

[B87] Tabatabai M. A. Bremner J. M. (1969). Use of p-nitrophenyl phosphate for assay of soil phosphatase activity. Soil Biol. Biochem. 1, 301–307. 10.1016/0038-0717(69)90012-1

[B88] Usero F. M. Armas C. Morillo J. A. Gallardo M. Thompson R. B. Pugnaire F. I. (2021). Effects of soil microbial communities associated to different soil fertilization practices on tomato growth in intensive greenhouse agriculture. Appl. Soil Ecol. 162, 103896. 10.1016/j.apsoil.2021.103896

[B89] Vance E. Brookes P. Jenkinson D. (1987). An extraction method for measuring soil microbial biomass C. Soil Biol. Biochem. 19, 703–707. 10.1016/0038-0717(87)90052-6

[B90] Venkatramanan V. Shah S. Rai A. K. Prasad R. (2021). Nexus between crop residue burning, bioeconomy and sustainable development goals over north-western India. Front. Energy Res. 8, 614212. 10.3389/fenrg.2020.614212

[B91] Wilkes T. I. Warner D. J. Edmonds-Brown V. Davies K. G. Denholm I. (2021). Zero tillage systems conserve arbuscular mycorrhizal fungi, enhancing soil glomalin and water stable aggregates with implications for soil stability. Soil Syst. 5, 4. 10.3390/soilsystems5010004

[B92] Wright S. F. Upadhyaya A. (1996). Extraction of an abundant and unusual protein from soil and comparison with hyphal protein of arbuscular mycorrhizal fungi. Soil Sci. 161, 575–586. 10.1097/00010694-199609000-00003

[B93] Xomphoutheb T. Jiao S. Guo X. Mabagala F. S. Sui B. Wang H.. (2020). The effect of tillage systems on phosphorus distribution and forms in rhizosphere and non-rhizosphere soil under maize (Zea mays L.) in Northeast China. Sci. Rep. 10, 6574. 10.1038/s41598-020-63567-7 32313140 PMC7171091

[B94] Yadvinder-Singh Bijay-Singh L. adha J. K. Khind C. S. Khera T. S. Bueno C. S. (2004). Effects of Residue Decomposition on Productivity and Soil Fertility in Rice–Wheat Rotation. Soil Sci. Soc. Am. J. 68, 854–864. 10.2136/sssaj2004.8540

[B95] Young J. C. (1995). Microwave-assisted extraction of the fungal metabolite ergosterol and total fatty acids. J. Agric. Food Chem. 43, 2904–2910. 10.1021/jf00059a025

[B96] Zhang P. Chen X. Wei T. Yang Z. Jia Z. Yang B.. (2016). Effects of straw incorporation on the soil nutrient contents, enzyme activities, and crop yield in a semiarid region of China. Soil Tillage Res. 160, 65–72. 10.1016/j.still.2016.02.00625880452

[B97] Zhang Z. Yan J. Han X. Zou W. Chen X. Lu X.. (2021). Labile organic carbon fractions drive soil microbial communities after long-term fertilization. Glob. Ecol. Conserv. 32, e01867. 10.1016/j.gecco.2021.e01867

[B98] Zuber S. M. Behnke G. D. Nafziger E. D. Villamil M. B. (2018). Carbon and Nitrogen Content of Soil Organic Matter and Microbial Biomass under Long-Term Crop Rotation and Tillage in Illinois, USA. Agriculture 8, 37. 10.3390/agriculture8030037

