# Supplementary material for: Influence of tillage and residue management practices on productivity, sustainability, and soil biological properties of rice-barley cropping systems in indo-gangetic plain of India
Source: Front Microbiol. 2023 Mar 16;14:1130397. doi: 10.3389/fmicb.2023.1130397 (PMC10060812; doi:10.3389/fmicb.2023.1130397)
Supplement: Supplementary file 1 [file Data_Sheet_1.docx]

Supplementary Table1: Grain yields of rice and barley during study years

| Treatments | Rice (t/ha) | | | | | | Barley (t/ha) | | | | | |
| --- | --- | --- | --- | --- | --- | --- | --- | --- | --- | --- | --- | --- |
|  | 2013 | 2014 | 2015 | 2016 | 2017 | Pooled | 2013-14 | 2014-15 | 2015-16 | 2016-17 | 2017-18 | Pooled |
| RTDSR–ZTB | 3.16 | 2.88 | 3.02 | 3.50 | 3.19 | 3.15 | 4.00 | 3.21 | 4.25 | 4.44 | 4.37 | 4.05 |
| RTDSR–ZTB–Gg | 3.20 | 3.00 | 3.12 | 3.60 | 3.28 | 3.24 | 4.06 | 3.40 | 4.25 | 4.48 | 4.45 | 4.13 |
| ZTDSR–ZTB–ZTGg | 3.15 | 2.95 | 3.27 | 3.63 | 3.35 | 3.27 | 3.98 | 3.30 | 4.31 | 4.58 | 4.59 | 4.15 |
| RTDSR–ZTB_RR4_ | 3.10 | 2.85 | 3.20 | 3.57 | 3.40 | 3.22 | 4.20 | 3.49 | 4.36 | 4.55 | 4.64 | 4.25 |
| RTDSR–ZTB_RR6_ | 3.18 | 2.90 | 3.25 | 3.58 | 3.46 | 3.27 | 4.32 | 3.60 | 4.42 | 4.58 | 4.67 | 4.32 |
| UPTR–ZTB–Gg | 3.45 | 3.26 | 3.45 | 3.80 | 3.62 | 3.52 | 4.00 | 3.32 | 4.23 | 4.48 | 4.53 | 4.11 |
| UPTR–ZTB_RR4_ | 3.49 | 3.30 | 3.40 | 3.75 | 3.60 | 3.51 | 4.30 | 3.53 | 4.32 | 4.54 | 4.63 | 4.26 |
| UPTR–ZTB_RR6_ | 3.50 | 3.35 | 3.49 | 3.76 | 3.60 | 3.54 | 4.32 | 3.54 | 4.39 | 4.55 | 4.67 | 4.29 |
| PTR–RTB | 3.41 | 3.41 | 3.65 | 3.81 | 3.79 | 3.61 | 3.98 | 3.16 | 4.19 | 4.37 | 4.38 | 4.02 |

RTDSR–ZTB: reduced till direct seeded rice–zero till barley, RTDSR–ZTB–Gg: reduced till direct seeded rice–zero till barley–green gram, ZTDSR–ZTB–ZTGg: zero till direct seeded rice–zero till barley–zero till green gram, RTDSR–ZTBRR4: reduced till–direct seeded rice–zero till barley + rice residue at 4 t ha^‑1^, RTDSR–ZTBRR6: reduced till direct seeded rice–zero till barley + rice residue at 6 t ha^-1^, UPTR–ZTB–Gg: un-puddled transplanted rice–zero till barley–green gram, UPTR–ZTBRR4: un-puddled transplanted rice–zero till barley + rice residue 4 t ha^-1^, UPTR–ZTBRR6: un-puddled transplanted rice–zero till barley + rice residue 6 t ha^-1^, PTR–RTB: puddled transplanted rice– reduced till barley, R: residue.

**Supplimentary Figure 1.** Monthly average values of weather parameters during January 2015 to December 2017


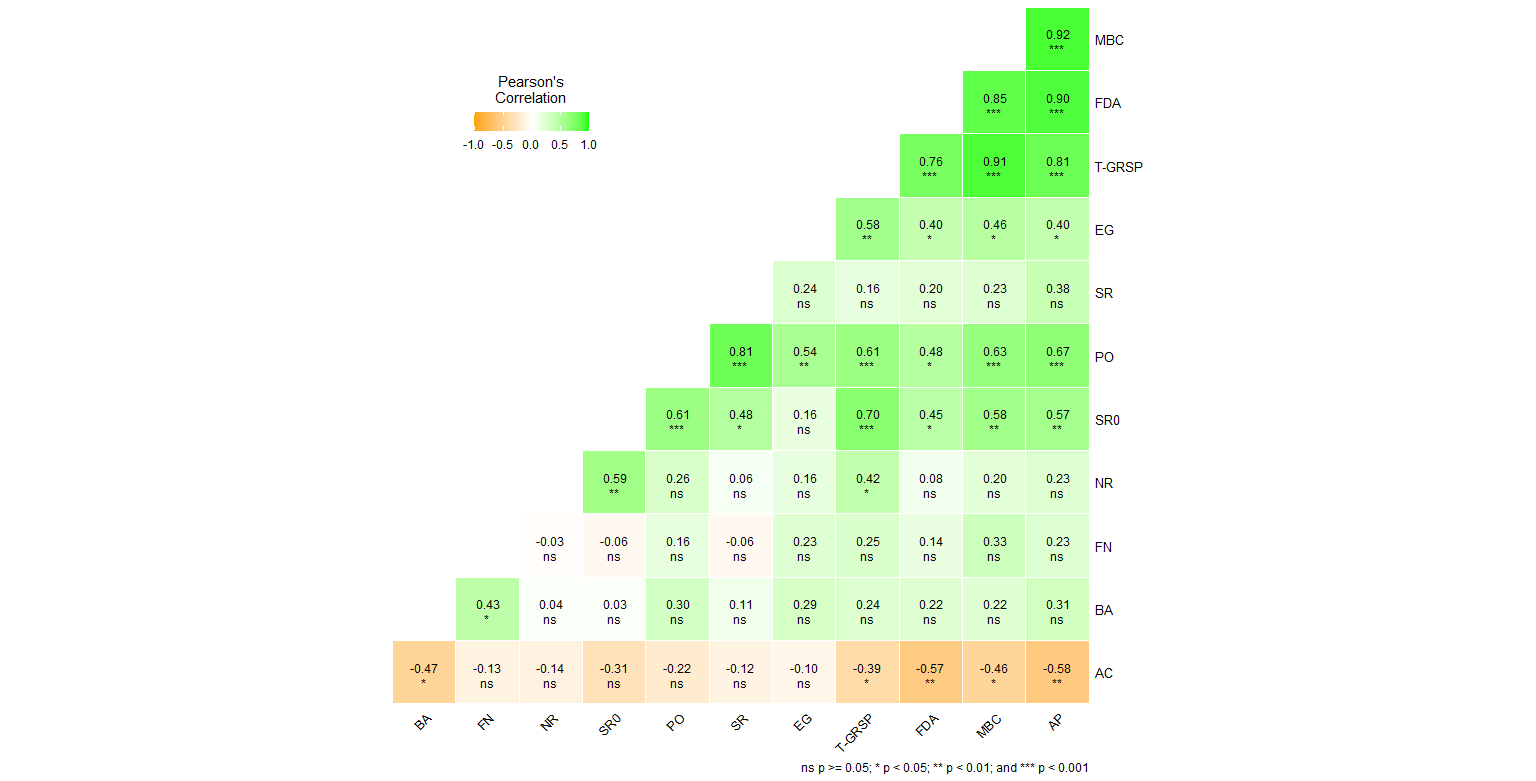


**Supplementary Figure 2:** Pearson’s correlation matrix among different soil biological parameters. (BA: Bacteria; AC: Actinobacteria; FN: Fungi; NR: Nitrate reductase activity; SR0:Soil respiration; PO: Peroxidase ;SR: Glucose induced soil respiration; EG: Ergosterol; T-GSRP: Total glomalin related soil proteins; FDA: Fluorescein diacetate hydrolysis; MBC: Microbial biomass Carbon; AP : Alkaline phosphatase)
